# An end-to-end approach for single-cell infrared absorption spectroscopy of bacterial inclusion bodies: from AFM-IR measurement to data interpretation of large sample sets

**DOI:** 10.1186/s12951-024-02674-3

**Published:** 2024-07-10

**Authors:** Wouter Duverger, Grigoria Tsaka, Ladan Khodaparast, Laleh Khodaparast, Nikolaos Louros, Frederic Rousseau, Joost Schymkowitz

**Affiliations:** 1 Switch Laboratory, VIB-KU Leuven Center for Brain and Disease Research, Herestraat 49, Leuven, 3000 Belgium; 2 Switch Laboratory, Department of Cellular and Molecular Medicine, KU Leuven, Herestraat 49, Leuven, 3000 Belgium; 3 Laboratory for Neuropathology, Department of Imaging and Pathology, KU Leuven, Herestraat 49, Leuven, 3000 Belgium; 4 Leuven Brain Institute, KU Leuven, Herestraat 49, Leuven, 3000 Belgium; 5 Center for Alzheimer’s and Neurodegenerative Diseases, Peter O’Donnell Jr. Brain Institute, University of Texas Southwestern Medical Center, Dallas, TX 75390 USA; 6 Department of Biophysics, University of Texas Southwestern Medical Center, Dallas, TX 75390 USA

**Keywords:** AFM-IR, Image analysis, Protein aggregation, *Escherichia coli*, Infrared spectroscopy

## Abstract

**Background:**

Inclusion bodies (IBs) are well-known subcellular structures in bacteria where protein aggregates are collected. Various methods have probed their structure, but single-cell spectroscopy remains challenging. Atomic Force Microscopy-based Infrared Spectroscopy (AFM-IR) is a novel technology with high potential for the characterisation of biomaterials such as IBs.

**Results:**

We present a detailed investigation using AFM-IR, revealing the substructure of IBs and their variation at the single-cell level, including a rigorous optimisation of data collection parameters and addressing issues such as laser power, pulse frequency, and sample drift. An analysis pipeline was developed tailored to AFM-IR image data, allowing high-throughput, label-free imaging of more than 3500 IBs in 12,000 bacterial cells. We examined IBs generated in *Escherichia coli* under different stress conditions. Dimensionality reduction analysis of the resulting spectra suggested distinct clustering of stress conditions, aligning with the nature and severity of the applied stresses. Correlation analyses revealed intricate relationships between the physical and morphological properties of IBs.

**Conclusions:**

Our study highlights the power and limitations of AFM-IR, revealing structural heterogeneity within and between IBs. We show that it is possible to perform quantitative analyses of AFM-IR maps over a large collection of different samples and determine how to control for various technical artefacts.

**Supplementary Information:**

The online version contains supplementary material available at 10.1186/s12951-024-02674-3.

## Background

Inclusion bodies (IBs) are insoluble nonmembranous organelles in bacterial cells that store misfolded and aggregated proteins, first observed by Prouty et al. in recombinant bacteria [1, 2]. These structures have attracted significant research attention due to their strong regulation by the host proteostasis machinery and their association with cellular senescence [3–5]. The challenge of resolubilising IBs often arises during the scale-up of protein production processes; however, in certain instances, proteins within IBs may retain some degree of their native structure and catalytic activity, negating the necessity for resolubilisation [6]. Moreover, IBs are being explored as putative drug delivery systems owing to their cell permeability and controlled drug release kinetics [7, 8]. Additionally, cellular stressors such as starvation, senescence, and exposure to antibiotics can induce IB formation [9].

A wide range of techniques is employed to study the structural properties of IBs. Various techniques, including X-ray diffraction (XRD), Fourier transform infrared (FTIR) and Raman spectroscopy, nuclear magnetic resonance spectroscopy (NMR) and dye binding assays using Congo red, thioflavin T, thioflavin S, and pFTAA, have revealed that IBs possess amyloid-like characteristics [9–11]. Amyloid-like fibrils have been observed by transmission electron microscopy (TEM) and atomic force microscopy (AFM) upon digestion by proteinase K or trypsin [12]. The interactions of IBs with the proteostasis system and their dynamic behaviour have predominantly been studied using biochemical assays, as well as brightfield and fluorescence microscopy [4, 13–15].

Micro-FTIR (µFTIR) is one of the few methods that offers label-free direct imaging of the secondary structure of proteins in IBs that does not depend on their extraction [16]. Proteins mainly absorb IR light in two regions of the IR spectrum: the amide I band (1600–1700 cm^-1^) and the amide II band (1500–1600 cm^-1^). The former is sensitive to the secondary conformation of a protein: β-sheets absorb between 1620 and 1640 cm^-1^ and 1674–1700 cm^-1^, depending on their nature, while α-helices and disordered regions absorb around 1654 cm^-1^ and β-turns around 1672 cm^-1^ [17]. However, the resolution of µFTIR cannot exceed 2.5 μm, the Abbe limit at these wavelengths [18].

There has been an exceptional boom in infrared imaging methods for achieving higher resolution, such as optical photothermal infrared microscopy (OPTIR) and AFM-based methods such as atomic force microscopy-based infrared spectroscopy (AFM-IR) [19, 20], tip-enhanced Raman scattering (TERS) and scanning near-field optical microscopy (SNOM) [21]. Each of these methods has its merits and limitations; see Dazzi and Prater (2016) for a comparison of AFM-IR, TERS, and SNOM [18]. In this work, we attempted to develop a protocol for the study of bacterial IBs using AFM-IR.

AFM-IR relies on the thermal expansion of molecules upon illumination with IR light of a wavenumber matching internal vibrations in those molecules and is therefore also known as photothermal infrared microscopy (PTIR) [22]. While the illumination laser remains diffraction-limited, a sharp AFM probe is used for the infrared absorption readout, resulting in a lateral resolution as low as 10 nm [23]. The amplitude of photothermal expansion is often considered proportional to the FTIR spectrum but is also influenced by factors including the probe shape, incident laser power, quality of mechanical contact, etc. [24, 25], resulting in slight band shifts in comparison to traditional FTIR in regard to protein conformational analysis [24, 26, 27]. Since the invention of AFM-IR, many improvements have been made to this method, such as resonance-enhanced [28], tapping [29], surface-sensitive [30], and null-deflection AFM-IR [31]. Another line of research attempts to perform AFM-IR in water [32, 33],

Previous studies on bacteria have utilised AFM-IR to study DNA [34–37], biopolymer-producing species [38, 39], antibiotic resistance [40] or bacterial functional amyloids [41], primarily after depositing dried bacteria on a substrate [42]. AFM-IR has been shown to be capable of measuring changes in the cell wall composition that confer antibiotic resistance [40], visualising individual viruses injecting their genome into a cell [35], and studying bacterial functional amyloids [41]. Building on this line of research, in this work, we attempt to study the structural and temporal differences between IBs formed under different stress conditions by applying AFM-IR to explore variations in protein secondary structure in situ within bacterial cells. We provide a detailed protocol optimisation and the development of an end-to-end data analysis pipeline to support large-scale quantitative measurements of parameters in a single-cell and single-particle (IB) fashion. We show that the unprecedented sample size produced in our study overcomes the technical and biological variability of such challenging samples and conclude that AFM-IR is sensitive enough to detect IB formation in bacterial cells and to distinguish IBs arising from different stress conditions.

## Methods

### Bacterial growth conditions

10 µL of bacteria (*E. coli* strains BW25113, BL21, or BL21 with pET15b-TEV-p53 plasmid [43]) from a 15% glycerol stock stored at − 80 °C were suspended in MHB medium (Fisher, 11,703,503), supplemented with ampicillin (100 µg/mL) if required. The cells were cultured overnight at 37 °C with shaking at 215 rpm.

### Stress application

For Figs. 3 and 4, BL21 (or BL21 pET15b) cultures were split over different tubes and washed with saline. They were then spiked with hydrogen peroxide (4 mM final concentration), nickel dichloride (100 µM final concentration), cobalt dichloride (100 µM final concentration), P2 (25 µg/mL), P33 (16 µg/mL), or, for p53 overexpression, IPTG (to 1 mM) and incubated for 1 h or 10 min in the case of P33.

For the experiment shown in Fig. 5, *E. coli* BW25113 cultures were split into different tubes. The tube corresponding to the longest recovery condition was incubated at 49 °C for 1 h, after which it was moved to 37 °C for one or two hours. The other tubes were moved between the two temperatures such that they spent the correct amount of time at 37 °C after the one-hour heat shock.

### AFM-IR sample preparation

All samples were spun down (2 min at 4300 × g), and the supernatant was replaced with 1.5 mL of saline solution (twice) before fixation in 0.5 mL of glutaraldehyde (2.5 vol% in 0.1 M Na-cacodylate buffer) and incubation for one hour at room temperature. Then, we performed three washes with cacodylate buffer (spinning down for 2 min at 12,100 × g) before secondary fixation in osmium tetroxide (1 vol% in cacodylate buffer) for 2 h. The samples were washed twice in cacodylate buffer and successively transferred to an ethanol series (30, 50, 70, 90, 100, 100, 100%), rotating at 4 °C for 10 min after each step. Then, they were resuspended twice in propylene oxide (Sigma, 82320) and rotated at 4 °C for 15 min.

The epoxy embedding was performed in three stages, first by resuspending in a 1:1 epoxy and propylene oxide mixture, supplementing 27 µL BDMA per 1 mL epoxy (Agar Scientific, AGR1031, hard formulation), and incubating for 1 h at 4 °C while rotating. Second, we resuspended the samples in a 2:1 mixture and left them to dry overnight. Finally, we transferred the samples to 100% epoxy resin, dried them at low vacuum for 4 h and cured them at 60 °C for 2 days. We sectioned the resin blocks to a thickness of 95 nm (Leica Ultracut UCT) and transferred the sections onto silicon wafers (Ted Pella, 16008), which were then glued to a sample disc (Bruker, SD-102 or Electron Microscopy Sciences 75010) using Reprorubber Thinpour (Reprorubber, 16116).

### AFM-IR data acquisition

All samples were measured at least one time in resonance-enhanced mode with a pulse rate around 900 kHz. They were imaged at least under illumination with 1625 and 1650 cm^-1^ light, and spectra were collected at least five IB, cytoplasm and epoxy locations (as estimated by visual inspection of an IR Amplitude map at 1625 cm^-1^) when possible. Collecting epoxy spectra during every measurement session allows us to check and correct for tip contaminations.

A gold-coated cantilever (Bruker, PR-EX-nIR2-10, *k* = 0.2 N/m, *f*_*0*_ = 13 kHz, *r* = 20–35 nm) was mounted in a nanoIR3 (Bruker) equipped with a MIRcat-QT laser (DRS Daylight Solutions), maximizing the laser sum, and adjusting the vertical and lateral deflection to approximately − 0.3 V and 0 V respectively. With the laser power set to 1.37%, a pulse rate of approximately 880 kHz and a pulse length of 160 ns, the IR beam was aligned in the x and y directions for each of the QCL chips (at 1730, 1260, 1088, and 914 cm^-1^), while its z position was optimised at 1730 cm^-1^. The atmospheric humidity is controlled by purging the system with dry air. Care is taken to let the relative humidity stably drop below 1% before measurements are made.

A phase offset was chosen to maximise the IR Amplitude, and the phase-locked loop (PLL) gains were set to *I* = 0.1 and *P* = 1. After collecting a laser emission spectrum (also called power spectrum or background spectrum), one IR spectrum was collected on epoxy to check that all parameters were set correctly. AFM-IR datasets were acquired at 1650 cm^-1^ and 1625 cm^-1^ with the following settings: field of view, 10 × 10 or 20 × 20 μm; resolution, 512 × 512 px (hence a pixel size of approximately 20–40 nm); scan rate, 0.1 Hz; AFM I gain, 2; P gain, 1; PLL I gain, 6; P gain, 60. For spectral measurements, we realigned the IR focus (only x and y), collected a new power spectrum, and collected spectra with the following settings: PLL I gain, 0.1; P gain, 1; spectral resolution, 2 cm^-1^; coaverages, 5; spectral range, 800–1800 cm^-1^; resolution, 2 cm^-1^. To change samples, we moved the sample to its lowest position and replaced it with a set of tweezers, taking care not to touch the AFM head.

### Data processing

The data were processed using python 3.10.13, numpy 1.26.3, pandas 2.1.4, SciPy 1.11.4, scikit-learn 1.3.2, scikit-image 0.19.3, statsmodels 0.14.1, umap-learn 0.5.5, xarray 2023.7.0, opencv-python-headless 4.9.0, and cellpose 2.2.3. Additionally, we adapted code developed by Dos Santos et al. [44].

IR absorption spectra, as reported, were normalised with respect to the laser emission spectrum at the time of measurement but were further processed by dividing them by the average epoxy spectra from the same sample measurement session and then min–max normalised to the range 0–1 between 1600 and 1800 cm^-1^. Every spectrum was reduced to a single PLL Frequency value by computing the average PLL Frequency between 1600 and 1650 cm^-1^ and subtracting the average epoxy PLL Frequency from the same sample and measurement session.

The AFM-IR datasets were processed as follows. First, the 1625 cm^-1^ IR Amplitude map was segmented into background, bacterium, and IB pixels. Cells are defined using a Cellpose model finetuned to our data and eroded to discard membrane pixels [45]. Cells intersecting the image border were discarded for analysis. Then, the intensity distribution of pixels inside a cell (IR Amplitude map at 1625 cm^-1^) was thresholded using the triangle algorithm, a binary opening was applied to discard noise pixels to obtain the IB map, and IB pixels outside of the cell mask were discarded [46]. Second, the IR Amplitude map at 1625 cm^-1^ was registered onto the 1650 cm^-1^ map to correct for sample drift. In the case of a constant drift, a simple translation would suffice, but nonconstant drift can introduce apparent image shearing. Therefore, registration is implemented in two steps, initially maximising the cross-correlation between the two matching height maps while allowing only rigid transformations and then allowing affine transformations. Finally, the PLL Frequency maps are processed to correct for PLL Frequency drift and cantilever variations by calculating the average PLL Frequency of epoxy pixels line-by-line, applying a rolling mean, and subtracting this profile from the whole map. Because of the higher IR amplitudes at 1650 cm^-1^, the PLL map corresponding to this wavelength was used in downstream analyses.

For the statistical analysis of multiple groups, Shapiro and Bartlett tests were performed to choose between ANOVA or Kruskal‒Wallis tests, after which suitable post hoc tests were performed. For multiple comparisons, p values were Bonferroni-corrected.

## Results

### Optimisation of data collection parameters

We started by optimising the data collection procedure, focusing on experimental parameters for AFM and those specific to AFM-IR such as the excitation laser power and pulse rate. Considering that optimal settings for a field of view measuring 10–20 μm wide necessitate slow scanning speeds [47], we quantified sample drift. Additionally, methods for plotting raw data from AFM-IR datasets were implemented for quality control purposes.

**Fig. 1:**
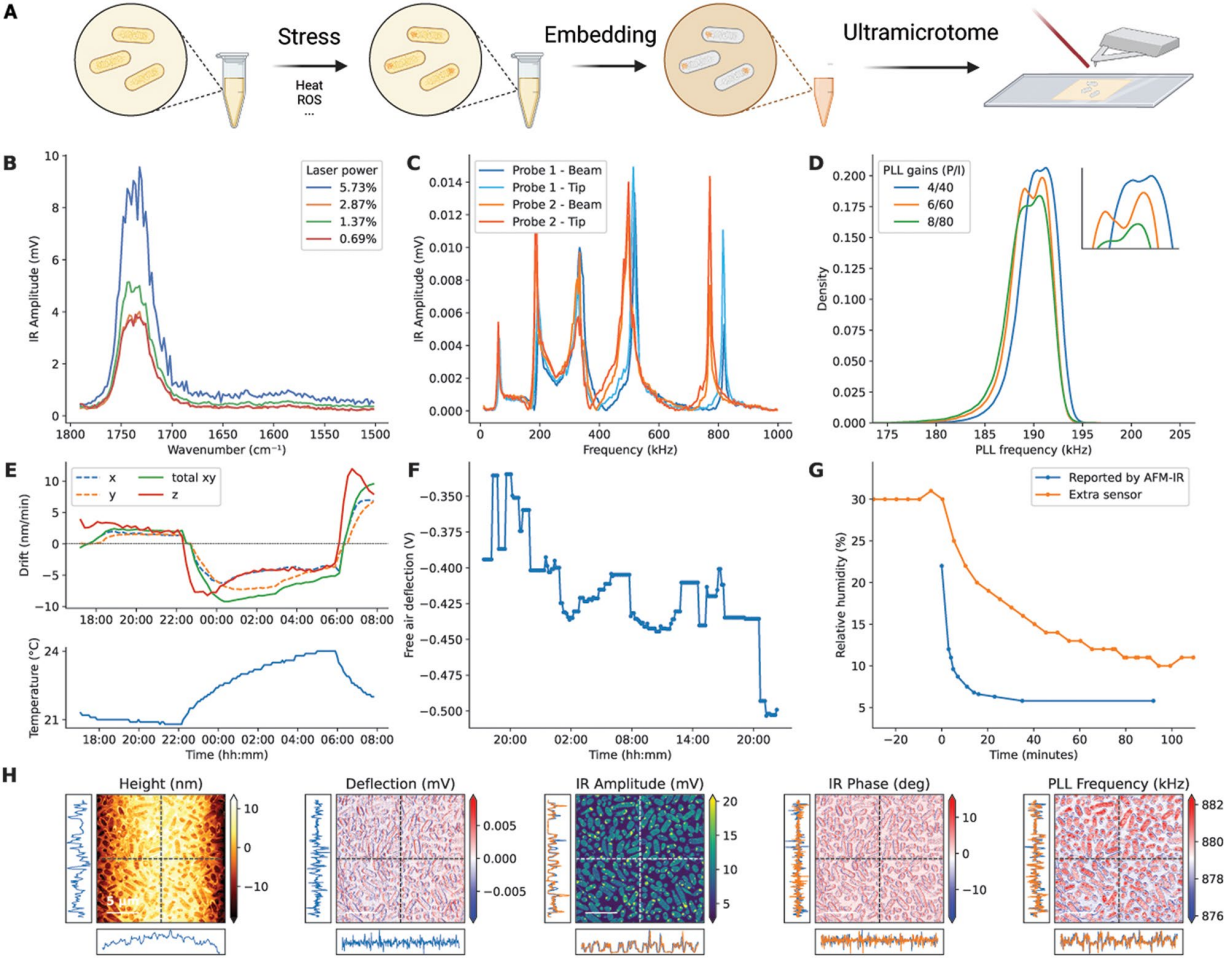
Protocol optimisation. **(A)** Schematic representation of the experimental protocol, created with Biorender.com. **(B)** IR Amplitude spectra of epoxy resin at various laser power settings. **(C)** Dependence of the IR Amplitude on the laser pulse rate varies from probe to probe and is influenced by the location at which the deflection laser hits the cantilever. **(D)** The distribution of values in a PLL Frequency map acquired under different feedback gain settings. The inset shows how the two different distributions in the sample (cells and epoxy) are most clearly separated at the 6/60 setting. **(E)** Measured drift speeds in the x, y, and z directions of the sample relative to the probe during an overnight measurement (top), correlated to the laboratory temperature (bottom). **(F)** Drift in the free-air deflection over the same period as (E). **(G)** Discrepancy between the reported and actual atmospheric humidity after opening the dry air purging valve at t = 0. **(H)** Output of the quality control pipeline for AFM-IR datasets showing maps and two data profiles in the trace (blue) and retrace (orange) scanning directions along the lines shown in the image

Bacterial cells were embedded in epoxy resin after fixation, and AFM-IR was conducted on 95 nm thick sections of the produced resin blocks (Fig. 1A). This embedding approach provides superior sample shelf life and surface smoothness, facilitating imaging [48]. At the point of loading the cantilever, we adjust the deflection mirror to ensure free-air deflection is about − 0.3 V, matching the default engagement force to achieve a deflection setpoint close to 0 V. The scan rate and AFM feedback gains were optimised to maintain a deflection within 0.01 V of the setpoint during a measurement, except around very sharp features such as knife marks.

To determine the common optimal laser power for all measurements, we collected spectra at various power levels at sample locations devoid of cells, as reflected by the absence of the protein-originating amide I band (Fig. 1B). The IR Amplitude signal at a laser power of 1.37% was larger than at both lower (0.69%) and higher (2.87%) power, indicating optimal field enhancement due to surface plasmon resonance [49]. At even higher power (5.73%), significant noise appeared around the absorption peak. Furthermore, it is recommended to avoid using excessively high laser powers, as this can potentially damage the sample. We conclude that 1.37% is the optimal power level for these samples on our system.

In resonance-enhanced AFM-IR, the repetition frequency of the IR laser needs to match the contact frequency of the sample-cantilever system [25], which varies from cantilever to cantilever but also depends on where the deflection laser hits the cantilever (Fig. 1C). The optimal frequency was tracked using a phase-locked loop (PLL), as it is subject to drift and contingent upon the nanomechanical properties of the sample and cantilever. Therefore, investigating the PLL Frequency maps reveals nanomechanical differences in the sample, although it cannot offer a direct quantification of the Young’s modulus [50, 51]. The gains of the PLL were determined through scanning experiments of epoxy-embedded bacteria. *I *= 6 and *P* = 60 provided the best separation between epoxy and bacteria (Fig. 1D). When acquiring a collection of spectra at various locations throughout the sample, we opt for low PLL gains (*I *= 0.1, *P* = 1) to reduce noise while allowing the PLL Frequency to adapt to slow changes in the optimal pulse rate.

Given the slow scanning speeds employed, sample drift may cause issues if left uncorrected. Temperature variations in the laboratory environment were found to exert a pronounced influence on sample drift relative to the AFM probe. Drift correction strategies were employed based on the observed drift patterns. This was done by collecting a series of height maps of the same sample, in this case, 2 × 2 μm height maps of amyloid protein on a gold substrate over a period of over 12 h (Fig. 1E; underlying data are presented in Supplementary Information, Note S1). Temperature-dependent drift is apparent both in the sample plane (x and y) and its vertical position (z). Based on our data, drift speeds on the order of 5–10 nm/min should be expected, even at relatively constant temperatures, and drift correction may be necessary [52].

Vertical sample drift was automatically compensated for by the AFM height tracking feedback. However, drift in the cantilever’s free air deflection requires additional consideration to ensure consistent force application during acquisition. Similar measurements over 2 days at near-constant temperatures (within 28 ± 0.2 °C) and an average sample drift of only 0.4 nm/min revealed differences in the free air deflection when automated deflection setpoint adjustment between acquired height maps was allowed (Fig. 1F). Given an engagement force of 0.3 V, these differences were large. As such, they required counteracting by resetting the deflection setpoint between map acquisitions; otherwise, this would result in strong variations in the force applied on the sample and cantilever and therefore the optimal pulse rate.

We assessed the accuracy of the humidity sensor in our system because atmospheric water vapour profoundly impacts IR spectra in the mid-infrared region due to its sharp absorption lines. While regular collection of the laser emission spectrum before each measurement partially compensates for this effect, periodic verification of the relative atmospheric humidity throughout an experiment is advisable, ideally maintaining levels below 1%. Notably, the placement of the humidity sensor in a nanoIR3 system near the supply of dry air may yield humidity readings that appear overly optimistic compared to readings obtained from a sensor positioned adjacent to the sample location (Fig. 1G). Thus, it is imperative to allow humidity levels to fully equilibrate before collecting IR measurements.

Finally, we acquired AFM-IR datasets at 1770 cm^-1^, a wavenumber at which no IR absorption is expected for epoxy or cells, to ensure that there is no IR Amplitude signal due to confounding mechanical effects. For this data, see Supplementary Information, Note S1.

Despite the implementation of these optimisations, the stability of the system may not always be sufficient to guarantee high-quality measurements. To ensure the integrity of our data, we acquired Height and Deflection maps in one scanning direction and IR Amplitude, IR Phase, and PLL Frequency maps in both scanning directions (trace and retrace) without applying any data processing. This approach enables the assessment of data quality both during and after measurement (Fig. 1H). Through this method, we can evaluate trace-retrace errors and assess the magnitude of deflection and IR phase signals, minimising deviations from zero. For all AFM-IR datasets and spectra published in this work, the raw data can be found in Supplementary Information, Note S2.

Throughout the rest of the paper, we will be using “AFM-IR dataset” for a set of images or maps with different types of data (Height, Deflection, IR Amplitude, PLL Frequency and IR Phase) collected simultaneously, and “IR Amplitude spectra” or simply “spectra” for IR absorbance spectra collected with the AFM-IR instrument.

### Data analysis pipeline and signal reproducibility

We established a pipeline for the automated analysis of AFM-IR datasets and spectra collected with the predefined parameters. Refer to the Methods section for details. To evaluate the performance of our measurement and analysis protocols, we prepared five identical samples of bacteria with spontaneous inclusion body (IB) formation and conducted multiple imaging sessions for each sample (*n* = 3–4), utilising the same cantilever whenever possible (refer to Supplementary Information, Note S3 for additional sample and cantilever details). This approach enabled us to assess both technical and biological variability.

**Fig. 2:**
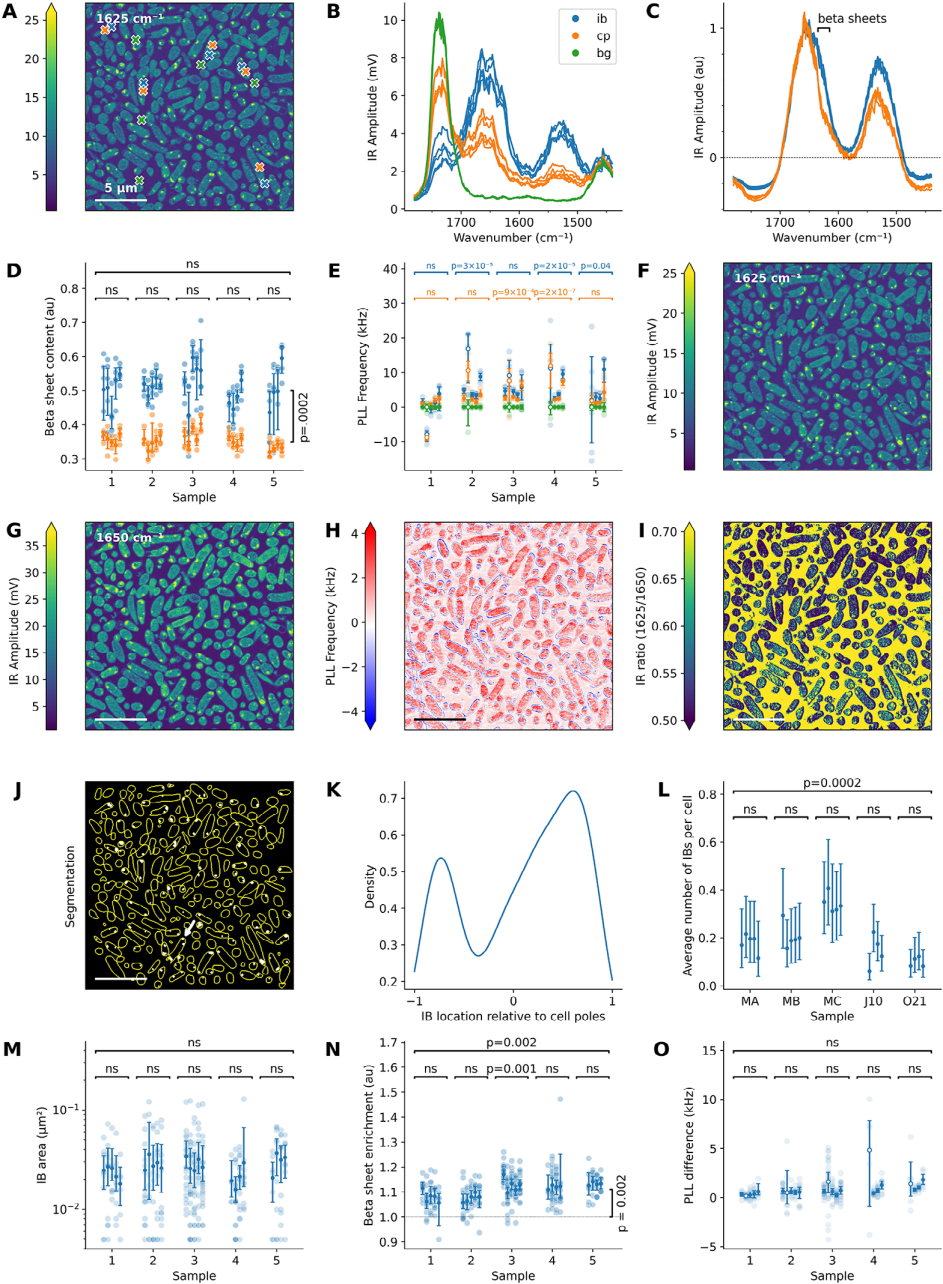
Data processing and analysis. **(A)** IR Amplitude map of thin section of bacteria embedded in epoxy and localisation of example spectra shown in (B). **(B)** IR Amplitude spectra after normalisation with respect to the laser power spectrum and **(C)** after further processing. The wavenumber range used for quantification of β-sheets (1615–1635 cm^-1^) is indicated. **(D)** Relative β-sheet content from IB (blue) and cytoplasm (orange) IR Amplitude spectra over five independent but biologically similar samples. Each column represents an independent measurement. Horizontal annotations indicate whether the data within contained groups with significantly different means. The vertical annotation highlights the significant difference between IB and cytoplasm β-sheet levels. **(E)** Quantification of the average PLL Frequency of these spectra, relative to the mean PLL Frequency of the epoxy spectra in that measurement session. Measurement 2, an outlier, is indicated by hollow markers. **(F)** Example of processed AFM-IR dataset, including an IR Amplitude map at 1625 cm^-1^, **(G)** an IR Amplitude map at 1650 cm^-1^, **(H)** a PLL Frequency map, **(I)** a ratio map of the IR Amplitudes, and **(J)** segmentation into cells and inclusion bodies based on the 1625 cm^-1^ IR Amplitude map. The white arrow highlights a cell with 3 segmented inclusion bodies. **(K)** Distribution of IBs along the cell major axis. **(L)** Plotted like (D), the average number of IBs per cell, **(M)** their area, **(N)** enrichment of their β-sheet ratio (average 1625/1650 ratio) relative to the cytoplasm of the same cell, and **(O)** their average PLL Frequence relative to the cytoplasm. Error bars represent a 95% confidence interval of the mean by bootstrap

In each individual measurement, we collected two AFM-IR datasets, one with illumination 1625 cm^-1^ (representing β-sheets [26]) and one at 1650 cm^-1^ (representing α-helices and unordered loops [26]), along with five IR spectra corresponding to inclusion bodies (IB), cytoplasm (CP), and epoxy (background; BG). Representative spectra and their locations are shown in Fig. 2A-C. For all spectra in this study, location data are provided in **Figure S2**. To quantify the relative β-sheet content in each spectrum, we integrated the area from 1615 to 1635 cm^-1^ (Fig. 2D). Our analysis revealed an enrichment of β-sheets in IBs compared to the cytoplasm. The observed β-sheet enrichment had a relative magnitude of 1.4 (95% CI: 1.36–1.52, two-sample t test: $p_{adj}=10^{-6}$). Notably, the technical variability observed did not yield statistically significant differences between repeat measurements (ANOVA on all data points for each sample: *p*_*adj*_ > 0.3). Moreover, no significant biological variability was observed (ANOVA on averages of each replicate: *p* > 0.76) in this assessment.

On the other hand, the PLL Frequency analysis (Fig. 2E) revealed significant technical variability (ANOVA on all data within each repeat: 9 > *p* _*adj*_ > 2 × 10^-5^), even after exclusion of an outlier measurement series (repeat 2, hollow markers). This technical variability masked any between-sample differences in the PLL Frequency of IBs, if there is any (ANOVA on averages of each replicate: *p*_*adj*_ = 2.2).

AFM-IR datasets provide a greater variety and depth of information than do spectra. They were first processed following the protocol detailed in the Methods section. Briefly, the pixels were classified as cell or background using a finetuned Cellpose model [45]. An IB map was generated by a binary threshold of the 1625 cm^-1^ IR Amplitude map, where the threshold was defined by the Triangle algorithm applied to the intensity histogram of the cell pixels, and a binary opening to discard noise pixels [46]. As a result, the smallest IBs detected have a radius of 2 pixels, corresponding to 40 nm or two times the nominal radius of the probe. Note that further experiments in this paper take a larger field of view, with twice the pixel size. However, since they are processed in the same manner, the smallest IBs will have a radius of 80 nm. This was done to increase throughput at the expense of resolution.

An example dataset is shown in Fig. 2F-J. For illustration purposes, this is a 20 × 20 μm dataset. The datasets underlying the analysis in this section can be consulted in Supplementary Information, Note S2. First, we observed polar enrichment of IBs (Fig. 2K); however, there were more IBs in the middle of the cell than expected from the literature [3]. This may be a result of the random three-dimensional orientation of cells with respect to the sectioning plane, but it is also possible that AFM-IR is sensitive to small protein aggregates that were not previously picked up by fluorescence microscopy approaches. Note that the relative age of the cell poles is not accessible in this experiment and that therefore, the sign of the polar location has no meaning. The positive pole is simply the one located on the right-hand side in the map.

Second, this dataset provides a measurement of the number of inclusion bodies per cell for each sample, as shown in Fig. 2L. Within this dataset, there was no significant technical variability (ANOVA on all data within each repeat: *p*_*adj*_ > 0.5), but biological variability (ANOVA on averages of each replicate: *p*_*adj*_ = 0.0002) was observed.

Third, this dataset contains a distribution of IB sizes (Fig. 2M), with an average radius of 85 nm, corresponding to eight pixels or four times the nominal radius of the AFM tip. There was no evidence of significant technical (ANOVA on all data within each repeat: *p*_*adj*_ > 10) or biological variability between the samples (ANOVA on averages of each replicate: *p*_*adj*_ = 0.5).

Fourth, the segmentation maps can be correlated to the IR Amplitude ratio and PLL maps to assess the physical and structural properties of IBs in an unbiased manner. Due to the inhomogeneous intensities of IR Amplitude maps discussed before, it is important to compare the relative β-sheet enrichment of an IB, the mean of the 1625/1650 cm^-1^ ratio map within the IB region, to that of the cytoplasm surrounding it (Fig. 2N). In this case, there was significant technical variability only within sample 3 (ANOVA on all data within sample 3: *p*_*adj*_ = 0.001, for other samples: *p*_*adj*_ > 9), but no biological variability between samples (ANOVA on averages of each replicate: *p*_*adj*_ = 0.06). We have not found the cause for this outlier measurement and can only recommend performing enough measurements so cases like these can be averaged out or discarded.

The relative β-sheet enrichment of inclusion bodies in this dataset was 1.11 (95% CI: 1.06–1.15, two-sample t test: *p*_*adj*_ = 0.0009). This enrichment value is lower than that measured in the spectral analysis, possibly because of the choice of wavenumbers for imaging.

Figure 2O shows the PLL Frequency difference between IBs and the surrounding cytoplasm. As in the spectral analysis, measurement 2 is an outlier. Excluding it, there was no statistical evidence for technical variability (ANOVA on all data within each repeat: *p*_*adj*_ > 0.1) or biological variability (ANOVA on averages of each measurement: *p*_*adj*_ > 1.2). While the PLL Frequency of IBs can be evaluated independently from the cytoplasm, this approach introduces extensive technical and biological variability (Supplementary Information, Note S4).

In summary, we developed a robust imaging pipeline providing data inaccessible by spectral analysis and independent of user bias due to the cherry-picking of spectrum locations. However, image analysis is limited by the discrete number of acquired wavenumbers and is more sensitive to technical artifacts, as shown in the ratio map in Fig. 2I.

### The nature of a stressor is reflected in the structure of resulting inclusion bodies

Having developed a robust imaging pipeline and evaluated its sensitivity to technical and biological variability, we attempted to distinguish IBs from various stress conditions by AFM-IR. A panel was selected to include physical stress (heat shock), chemical stress (heavy metals such as NiCl_2_, CoCl_2_ and oxidation by hydrogen peroxide) and proteotoxic stress (overexpression of the aggregation-prone p53 DNA-binding domain [43] or exposure to the peptides P2 and P33 [9]). Peptins are short hydrophobic peptides that nucleate the aggregation of endogenous proteins due to homology with aggregation-prone regions.

**Fig. 3:**
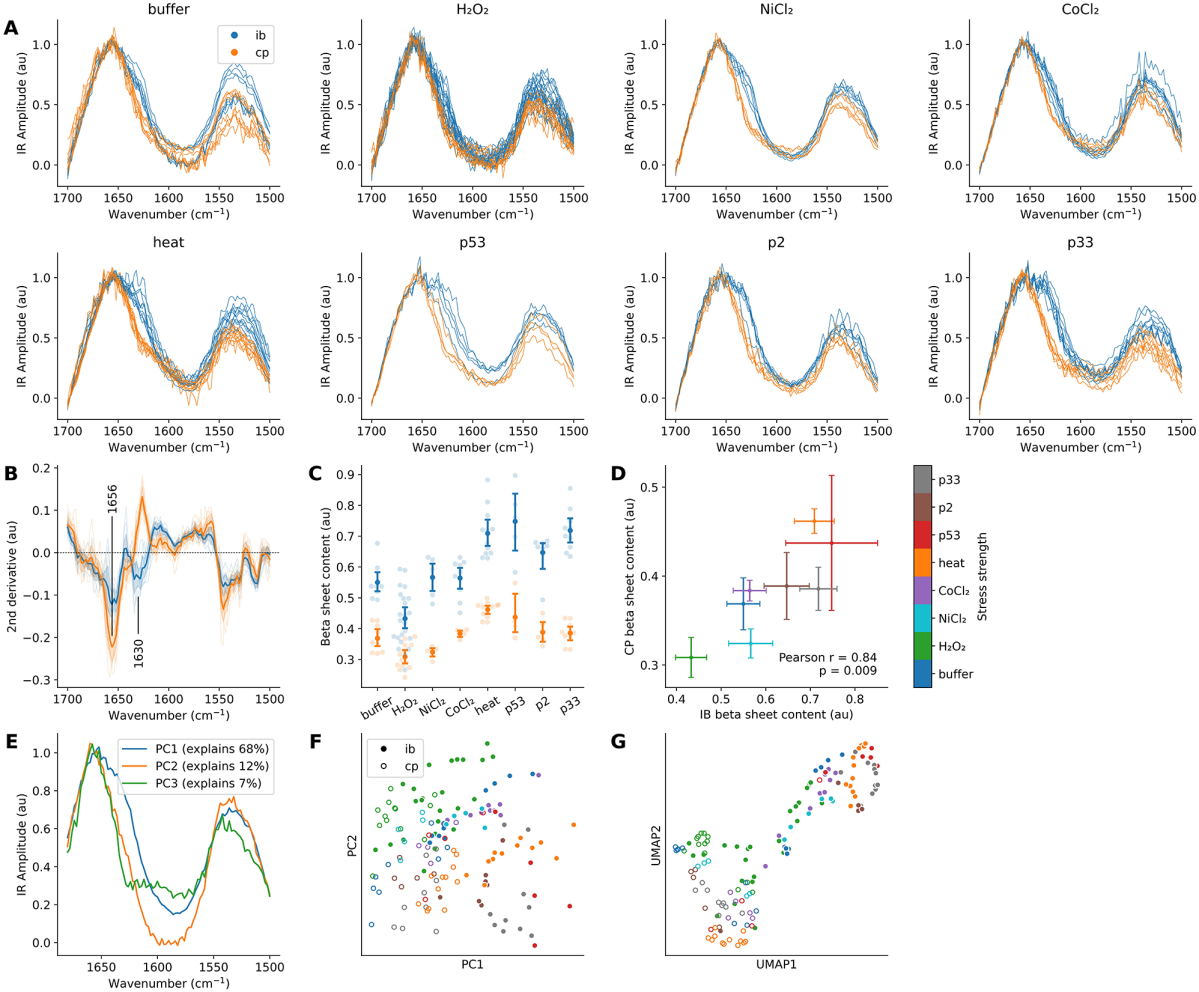
The nature of a stress affects the resulting IBs. **(A)** IR Amplitude spectra of IBs and cytoplasm collected from thin sections of epoxy-embedded bacteria after application of various stress conditions. **(B)** Second derivative spectra (averaged for each sample) display an increase in β-sheet content. The average over the whole dataset is represented by the mean and shaded CI (mean ± 1.96 × SEM; standard error of the mean). **(C)** Relative β-sheet content of IBs and cytoplasma in each spectrum. Mean and 95% CI (bootstrap). **(D)** Same as (C) but highlighting the correlation between cytoplasmic and IB β-sheet levels. Error bars represent mean ± 1.96 × SEM. **(E)** The first three principal components found in this data. **(F)** Score plot mapping all spectra to PCA space. The colours of the data points match panel (D). **(G)** UMAP representation of the spectral data

To increase the experimental throughput, only IR absorption spectra were collected for these samples, as shown in Fig. 3A. These experiments were performed in *E. coli* BL21 to accommodate the overexpression stress, but this strain also exhibited spontaneous IB formation in the buffer IB and cytoplasm were distinct from each other under all conditions, partly due to the increased β-sheet concentration, which was visible in the second derivative spectra (Fig. 3B). Figure 3C shows a quantification of the β-sheet content, the cytoplasmic levels of which were correlated with those in IBs (Pearson *r* = 0.84, 95% CI: 0.34-0.97, *p* = 0.009; Fig. 3D). Principle component analysis (PCA) indicated that the first principal component was highly sensitive to the β-sheet content (Fig. 3E). Both PCA and uniform manifold approximation and projection (UMAP) [53] could distinguish between the IB and cytoplasm spectra (Fig. 3F-G). Furthermore, IBs from heat shock and proteotoxic stress conditions formed a cluster, and the chemical stresses were intermediate between them and the cytoplasm spectra. In this sense, the AFM-IR spectra seem to reflect the severity and type of applied stress.

**Fig. 4:**
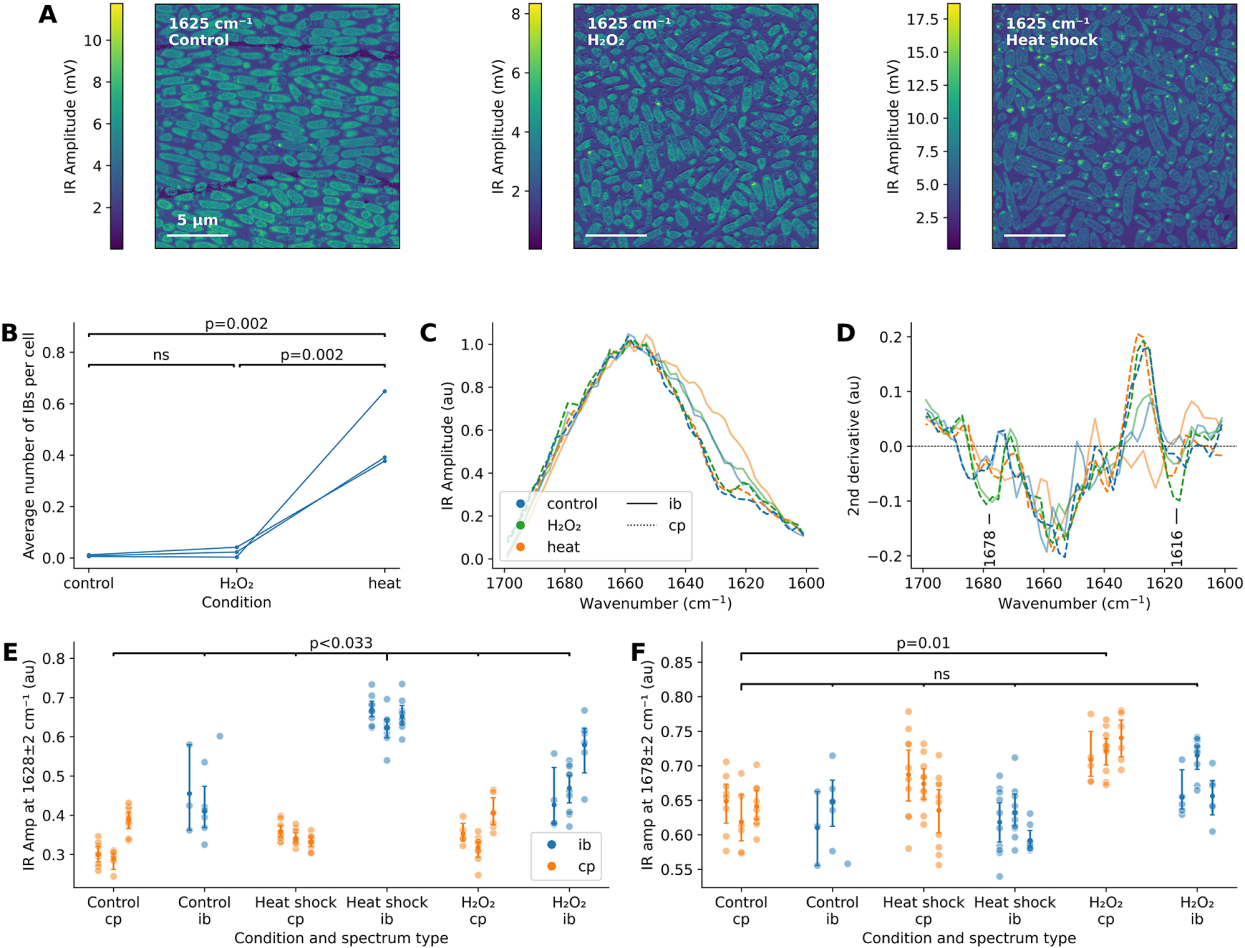
Validation of hydrogen peroxide stress. **(A)** Representative IR Amplitude maps of thin sections of bacteria embedded in epoxy resin after control, hydrogen peroxide, and heat shock treatment. **(B)** Number of IBs per cell. Compared to other conditions, heat shock causes much more IB formation (comparisons report p values from Tukey’s test). **(C)** Average IR Amplitude spectra of IBs and cytoplasma under the three conditions reveal differences in structural composition. **(D)** Averaged second derivative spectra from peroxide-treated bacteria are characterised by peaks at 1678 and 1616 cm^-1^. **(E)** Quantification of β-sheet levels (IR Amplitude intensity around 1628 cm^-1^). **(F)** Quantification of IR Amplitude intensity around 1678 cm^-1^. All spectra are plotted for each condition and replicate, and 95% CIs (bootstrap) are shown

Because these results were based on a single sample per condition, they needed to be validated. We therefore compared H_2_O_2_ stress to heat shock with a larger number of samples (*n* = 3) and full imaging following the protocol developed in this paper. Heat shock was shown to induce a much greater IB load (Fig. 4A, B). There were some inclusions visible in the hydrogen peroxide sample in Fig. 4A, but they were not recognised by the image segmentation pipeline, presumably due to their lower β-sheet enrichment and smaller size.

These smaller IBs could still be studied by collecting IR absorption spectra on locations that visually had a strong IR Amplitude signal at 1625 cm^-1^ (see Fig. 4C-D). Spectral analysis confirmed that heat shock IBs had the highest β-sheet content among all spectra quantified in Fig. 4E (Dunnett’s test: *p* < 0.033). Additionally, the second derivative spectra implied the existence of two new bands in the peroxide-stressed spectra at 1678 cm^-1^ (antiparallel β-sheets) and 1616 cm^-1^ (intermolecular β-sheets), although the latter was nearly invisible in the original spectra. The 1678 cm^-1^ band sets the peroxide cytoplasm spectra apart from all others (Fig. 4F): Dunnett’s test comparing all spectra to the control cytoplasm revealed no significant differences, except for the peroxide cytoplasm spectrum (*p*_*adj*_ = 0.01). We concluded that AFM-IR, at least in spectral mode, is sensitive enough to distinguish between different stresses based on the secondary structure of cytoplasmic and aggregated proteins in stressed cells.

### Recovery from heat shock

**Fig. 5:**
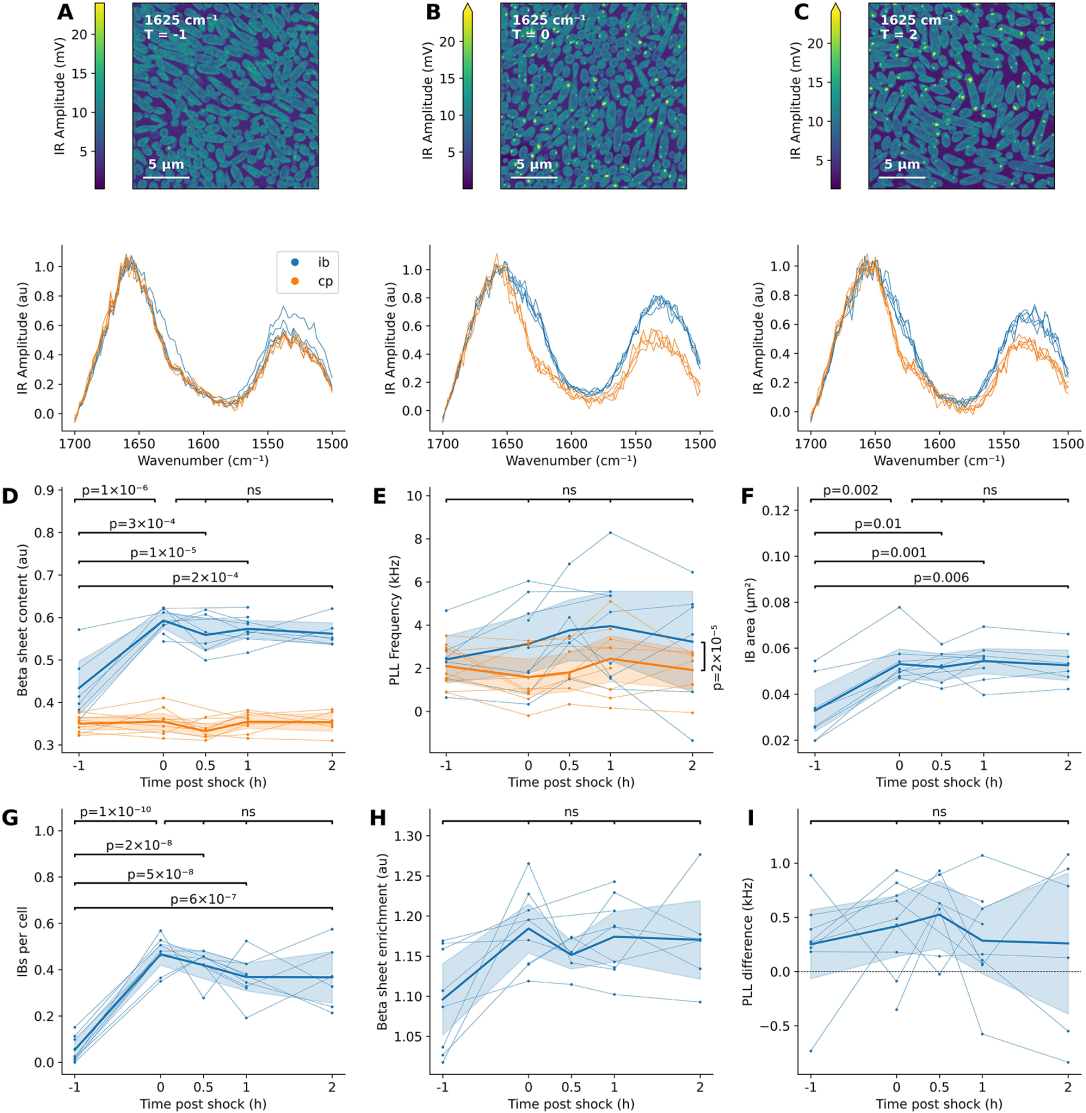
Recovery of IBs after heat shock. **(A)** Representative IR Amplitude maps (top) and spectra (bottom) of thin sections of bacteria embedded in epoxy resin after control treatment, **(B)** heat shock, **(C)** and heat shock with subsequent recovery, **(B)** immediately after heat shock and **(C)** two hours after heat shock. **(D)** Β-sheet content of inclusion bodies (integral of normalised spectra between 1615–1635 cm^-1^), averaged per sample. Biological replicates are connected by thin lines. Bold lines represent averages. **(E)** Average PLL Frequency of IB and cytoplasm IR Amplitude spectra, relative to epoxy. **(F)** The IB area increases during heat shock but remains constant afterwards, like **(G)** the number of IBs per cell and **(H)** their β-sheet enrichment. **(I)** IBs are more rigid than the cytoplasm at all timepoints tested, but there were no differences between timepoints. Shaded regions represent 95% CIs by bootstrap

To go even further, heat shock IBs were characterised in a time-resolved manner after returning to 37 °C (samples were collected before heat shock and immediately, 30 min, 1 h and 2 h after heat shock; Fig. 5A-C).

A quantification of the β-sheet signal from these spectra (Fig. 5D) showed that the IB spectra at all timepoints were significantly enriched in β-sheets compared to the IB spectra before heat shock (ANOVA followed by Tukey’s test: *p*_*adj*_ < 0.0003), but there was no evidence of significant changes in the β-sheet content during the recovery period (Tukey’s test: *p* > 0.6). The cytoplasmic β-sheet content was stable over time (ANOVA: *p* = 0.4). Due to the number of spectra in this experiment, it was possible to perform an accurate analysis of the second derivative spectra, which revealed the formation of both intramolecular and intermolecular β-sheets (Supplementary Information, Note S5). The PLL Frequency of IBs did not change over time between the IB spectra at different timepoints (ANOVA: *p* = 0.7), nor did cytoplasm spectra (ANOVA: *p* = 0.7, Fig. 5E). In general, however, IBs had a higher PLL Frequency than the cytoplasm of the same cell, reflecting their increased stiffness (Wilcoxon signed-rank test: *p*_*adj*_ = 2 × 10^-5^).

The image analysis data, specifically of the IB area (Fig. 5F) and number (Fig. 5G), showed similar trends: an increase during the heat shock with a steady state in the two hours afterwards. While the evolution of IB β-sheet enrichment was not statistically significant (ANOVA: *p*_*adj*_ = 0.1), its trend recapitulated the spectral quantification and remained significantly greater than 1 in general (95% CI: 1.13–1.18, two-sample t test: *p*_*adj*_  = 10^-18^, Fig. 5H). Similarly, the difference in PLL Frequency between IBs and the cytoplasm (Fig. 5I) did not vary over time (ANOVA: *p* = 0.8) but was positive (95% CI: 0.15-0.52, one-sample t test: *p*_*adj*_ = 0.0003).

In short, AFM-IR was unable to resolve any differences in the IB composition in the first two hours after heat shock. This could mean that disassembly takes longer than two hours under the conditions used in this paper [15], or it could be a limitation of the instrument. These data were validated by several orthogonal methods: the IBs were stained with the amyloid marker pFTAA and imaged using structured illumination microscopy to verify the amyloid nature of the β-sheets, one sample was imaged by transmission electron microscopy (TEM) and scanning electron microscopy (SEM) to electron density variations measure surface wear due to the AFM measurement, and IBs were purified and imaged by AFM-IR (Supplementary Information, Note S6).

### Using the full capabilities of AFM-IR

**Fig. 6:**
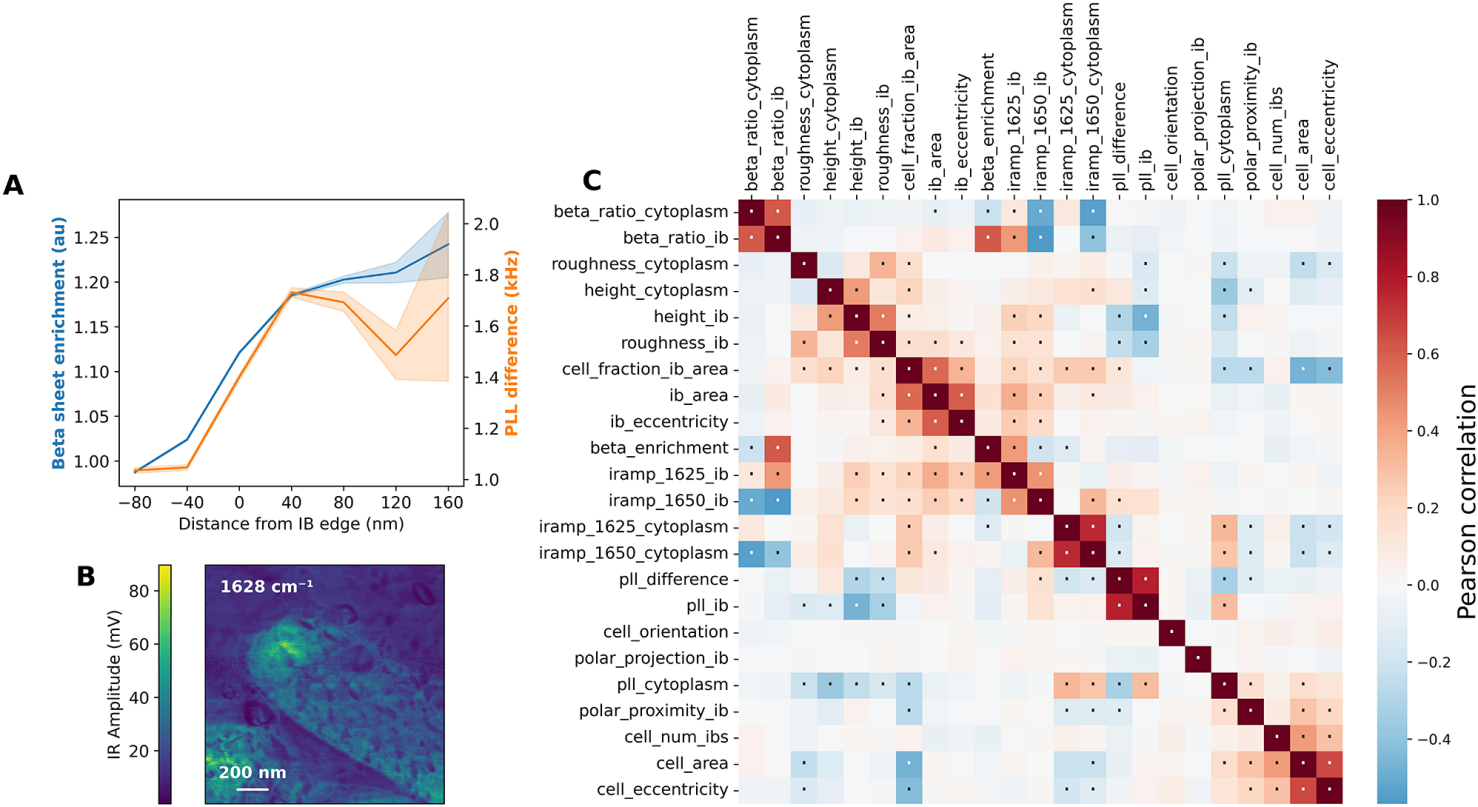
Highlighting the capabilities of image-based analysis of AFM-IR data. **(A)** Average β-sheet enrichment and PLL Frequency (relative to epoxy) of a pixel in an AFM-IR dataset as a function of its distance to the nearest IB edge (mean and 95% CI). **(B)** IR Amplitude map of an IB after heat shock treatment in a thin section of epoxy-embedded bacteria. **(C)** Correlation plot between IB properties. Dots highlight statistically significant correlations (Bonferroni-corrected *p* < 0.05)

The protocol presented in this paper sacrifices resolution in favour of faster acquisition times and larger fields of view, yet the resulting data did offer evidence that IBs are not sharply defined objects but that they have diffuse boundaries spanning approximately 120 nm (Fig. 6A). This figure shows the average β-sheet enrichment and PLL difference of all pixels in the heat shock recovery dataset as a function of their distance to the closest IB border, with negative values indicating pixels outside an IB. To substantiate this conclusion, we also present an example of the capabilities of the instrument at a sampling rate of approximately 1 pixel per 3 nm, as presented in Fig. 6B. This IR Amplitude map clearly shows a heterogeneous IB with diffuse edges.

In addition to the β-sheet content and PLL Frequency of each IB, a large set of other properties was measured, such as localisation, size and shape, thickness, etc. Some of these were found to be intimately connected with each other (Fig. 6C; see Supplementary Information, Note S7 for descriptions of each property). For this figure, Pearson correlations were calculated between all pairs of properties in the set of IBs in each of the AFM-IR datasets underlying Fig. 5. Bootstrap resampling (*n* = 9999) of the resulting set of correlations was used to test which ones are significantly different from 0.

As expected, neither cell orientation nor the polar projection of an IB is correlated with any other variable in this dataset. However, its proximity to a cell pole is part of a cluster of correlated variables likely driven by apparent cell size, which in turn is strongly dependent on the orientation of the cell with respect to the sectioning plane.

Somewhat unexpectedly, the relative β-sheet enrichment of an IB was largely uncorrelated to variables related to PLL Frequency and therefore stiffness. For reasons outlined earlier in this paper, we consider the difference in PLL a more robust readout than the mean IB PLL itself. The fact that the former does not correlate with β-sheet concentration (beta_ratio_ib) may mean that stiffness is driven by protein density than secondary structure, or it may reflect a lack of sensitivity to the small differences in β-sheet concentration and PLL Frequency within the set of measured IBs, even if it is established that IBs as a whole have a higher PLL than the cytoplasm. Furthermore, the correlation between PLL Frequency and local section thickness may additionally confound these observations. Finally, β-sheet enrichment was correlated with IB area and a cluster of definitionally related variables, such as the IR Amplitude at 1652 and 1650 cm^-1^. Even if our conclusions from this correlation analysis are limited, the analysis itself does show the potential of image-based AFM-IR experiments.

## Discussion

This paper describes the development of a protocol for performing high-throughput single-cell AFM‑IR spectroscopy on bacterial IBs. In total, this paper studies AFM-IR datasets at two wavenumbers of 12,030 cells, containing 3539 IBs, as well as 1343 spectra. Datasets of this size require saving all data in their rawest form possible, not only to evaluate the data quality but also to perform end-to-end automated data analysis, as developed in this paper. This means that our primary data are easily auditable and that our analysis is fully reproducible.

The scale of this dataset made it possible to, for the first time, make a rigorous assessment of data variability introduced by repeated measurements or biological variation. For most data outputs, the differences between repeated measurements were not significant, except for the PLL Frequency, which was found to be very sensitive to technical variability. Considerable biological variability between different samples was also observed, which is important for quantitative measurements.

Improving the stability of the PLL feedback system will be critical for robust assessments of nanomechanical heterogeneities in a correlative fashion with chemical and structural information derived from AFM-IR. For the moment, this need may be better served by AFM modes specifically developed for mechanical characterisation and not by using the PLL Frequency as a primary read out [54]. Even on systems with both AFM-IR and specific nanomechanical mapping modes, improved PLL stability will benefit the quality of the IR Amplitude signal. Users of a nanoIR3 system should attempt to minimise exogenous factors such as environmental noise, temperature fluctuations, power supply stability, and to make sure the system is fully equilibrated before initiating key measurements.

It was established that AFM-IR can detect differences between a set of various stresses, both in spectral and imaging mode, but cannot discern any evolution in IB properties over a two-hour recovery period after heat shock, revealing both the possibilities and the limitations of the method’s sensitivity. However, given its severity, the time allowed for recovery from heat shock was quite short.

Currently, the main limitations of AFM-IR lie in the long measurement times for IR absorption images and in the technical artefacts that can cause misinterpretations of the data. Acquiring one high-quality dataset can easily take three to four hours. PLL tracking of the IR pulse frequency is a strength and a limitation of this study: it offers mechanical information about the sample, but the PLL feedback can be unstable and lose tracking; therefore, PLL Frequency is the least reproducible output modality. While sections of epoxy-embedded samples provide smooth samples and faster imaging, the epoxy masks some regions of the IR spectrum, precluding a measurement of the IR response of lipids and nucleic acids. Fixing the samples prevents live time-lapse imaging, but this is already prohibited by the long scanning times. Additionally, it is unlikely to find entire cells in a field of view because of the random orientation of bacteria with respect to the sectioning plane. It would be interesting to perform image-based analyses on bacteria spotted directly on a substrate to circumvent the problems caused by epoxy embedding, although we anticipate additional imaging difficulties caused by the increased surface topology [42]. Efforts are underway to enable AFM-IR imaging in a liquid environment, which would open the door to live-cell imaging [55, 56].

AFM-IR has already been applied in medical contexts, for example to study drug uptake and formulation, protein aggregation in situ and in vitro, parasitic infections, and more [57–65]. We expect that improving technology and increasing ease-of-use of AFM-IR will enable even more biological applications of this method.

## Conclusion

We studied IB formation and recovery under heat shock and other stresses by rigorously optimising the data collection protocols and developing an imaging pipeline to process large datasets. This study shows the potential of AFM-IR for single-cell spectroscopy of large numbers of cells and IBs, details a method that could be applied to many questions in microbiology, and improves upon existing data analysis workflows using fully open-source software. Furthermore, the code published alongside this work should facilitate future analyses of large AFM-IR datasets and improve the transparency and reproducibility of data reported in this field.

### Electronic supplementary material

Below is the link to the electronic supplementary material.

Supplementary Material 1

## Data Availability

The data and code underlying this study are openly available on GitHub at https://github.com/wduverger/ib_spectroscopy and deposited in figshare at 10.6084/m9.figshare.25398622.v2.

## References

[1] Prouty WF, Karnovsky MJ, Goldberg AL (1975). Degradation of abnormal proteins in Escherichia coli. Formation of protein inclusions in cells exposed to amino acid analogs. J Biol Chem.

[2] Schramm FD, Schroeder K, Jonas K (2020). Protein aggregation in bacteria. FEMS Microbiol Rev.

[3] Lindner AB, Madden R, Demarez A, Stewart EJ, Taddei F (2008). Asymmetric segregation of protein aggregates is associated with cellular aging and rejuvenation. Proc Natl Acad Sci U S A.

[4] Carrio MM, Villaverde A (2003). Role of molecular chaperones in inclusion body formation. FEBS Lett.

[5] Stewart EJ, Madden R, Paul G, Taddei F (2005). Aging and death in an organism that reproduces by morphologically symmetric division. PLoS Biol.

[6] Peternel S, Grdadolnik J, Gaberc-Porekar V, Komel R (2008). Engineering inclusion bodies for non denaturing extraction of functional proteins. Microb Cell Fact.

[7] Unzueta U, Cespedes MV, Sala R, Alamo P, Sanchez-Chardi A, Pesarrodona M (2018). Release of targeted protein nanoparticles from functional bacterial amyloids: a death star-like approach. J Control Release.

[8] Villaverde A, Garcia-Fruitos E, Rinas U, Seras-Franzoso J, Kosoy A, Corchero JL (2012). Packaging protein drugs as bacterial inclusion bodies for therapeutic applications. Microb Cell Fact.

[9] Khodaparast L, Khodaparast L, Gallardo R, Louros NN, Michiels E, Ramakrishnan R (2018). Aggregating sequences that occur in many proteins constitute weak spots of bacterial proteostasis. Nat Commun.

[10] Wang L, Maji SK, Sawaya MR, Eisenberg D, Riek R (2008). Bacterial inclusion bodies contain amyloid-like structure. PLoS Biol.

[11] Pouplana S, Espargaro A, Galdeano C, Viayna E, Sola I, Ventura S (2014). Thioflavin-S staining of bacterial inclusion bodies for the fast, simple, and inexpensive screening of amyloid aggregation inhibitors. Curr Med Chem.

[12] Garcia-Fruitos E, Gonzalez-Montalban N, Morell M, Vera A, Ferraz RM, Aris A (2005). Aggregation as bacterial inclusion bodies does not imply inactivation of enzymes and fluorescent proteins. Microb Cell Fact.

[13] Carrio MM, Villaverde A (2005). Localization of chaperones DnaK and GroEL in bacterial inclusion bodies. J Bacteriol.

[14] Morell M, Bravo R, Espargaro A, Sisquella X, Aviles FX, Fernandez-Busquets X (2008). Inclusion bodies: specificity in their aggregation process and amyloid-like structure. Biochim Biophys Acta.

[15] Govers SK, Mortier J, Adam A, Aertsen A (2018). Protein aggregates encode epigenetic memory of stressful encounters in individual Escherichia coli cells. PLoS Biol.

[16] Ami D, Natalello A, Taylor G, Tonon G, Maria Doglia S (2006). Structural analysis of protein inclusion bodies by Fourier transform infrared microspectroscopy. Biochim Biophys Acta.

[17] Goormaghtigh E, Cabiaux V, Ruysschaert J-M, Hilderson HJ, Ralston GB (1994). Determination of Soluble and membrane protein structure by Fourier Transform Infrared Spectroscopy. Physicochemical methods in the study of Biomembranes.

[18] Dazzi A, Prater CB (2017). AFM-IR: technology and applications in Nanoscale Infrared Spectroscopy and Chemical Imaging. Chem Rev.

[19] Zhang D, Li C, Zhang C, Slipchenko MN, Eakins G, Cheng JX (2016). Depth-resolved mid-infrared photothermal imaging of living cells and organisms with submicrometer spatial resolution. Sci Adv.

[20] AC VDDS, Hondl N, Ramos-Garcia V, Kuligowski J, Lendl B, Ramer G (2023). AFM-IR for Nanoscale Chemical characterization in Life sciences: recent developments and future directions. ACS Meas Sci Au.

[21] Xitian H, Li Z, Xu W, Yan P (2023). Review on near-field detection technology in the biomedical field. Adv Photonics Nexus.

[22] Dazzi A, Prazeres R, Glotin F, Ortega JM (2005). Local infrared microspectroscopy with subwavelength spatial resolution with an atomic force microscope tip used as a photothermal sensor. Opt Lett.

[23] Schwartz JJ, Pavlidis G, Centrone A (2022). Understanding Cantilever transduction efficiency and spatial resolution in Nanoscale Infrared Microscopy. Anal Chem.

[24] Ramer G, Aksyuk VA, Centrone A (2017). Quantitative Chemical Analysis at the Nanoscale using the Photothermal Induced Resonance technique. Anal Chem.

[25] Quaroni L (2020). Understanding and Controlling spatial resolution, sensitivity, and Surface Selectivity in Resonant-Mode Photothermal-Induced Resonance Spectroscopy. Anal Chem.

[26] Waeytens J, De Meutter J, Goormaghtigh E, Dazzi A, Raussens V (2023). Determination of secondary structure of proteins by Nanoinfrared Spectroscopy. Anal Chem.

[27] Waeytens J, Mathurin J, Deniset-Besseau A, Arluison V, Bousset L, Rezaei H (2021). Probing amyloid fibril secondary structures by infrared nanospectroscopy: experimental and theoretical considerations. Analyst.

[28] Lu F, Belkin MA (2011). Infrared absorption nano-spectroscopy using sample photoexpansion induced by tunable quantum cascade lasers. Opt Express.

[29] Wieland K, Ramer G, Weiss VU, Allmaier G, Lendl B, Centrone A (2019). Nanoscale chemical imaging of individual chemotherapeutic cytarabine-loaded liposomal nanocarriers. Nano Res.

[30] Wang L, Wang H, Wagner M, Yan Y, Jakob DS, Xu XG (2017). Nanoscale simultaneous chemical and mechanical imaging via peak force infrared microscopy. Sci Adv.

[31] Kenkel S, Mittal S, Bhargava R (2020). Closed-loop atomic force microscopy-infrared spectroscopic imaging for nanoscale molecular characterization. Nat Commun.

[32] Mathurin J, Deniset-Besseau A, Dazzi A (2020). Advanced Infrared Nanospectroscopy using Photothermal Induced Resonance technique, AFMIR: New Approach using Tapping Mode. Acta Phys Pol A.

[33] Yilmaz U, Sam S, Lendl B, Ramer G (2024). Bottom-illuminated Photothermal Nanoscale Chemical Imaging with a Flat Silicon ATR in Air and Liquid. Anal Chem.

[34] Dazzi A, Prazeres R, Glotin F, Ortega JM (2006). Subwavelength infrared spectromicroscopy using an AFM as a local absorption sensor. Infrared Phys Technol.

[35] Dazzi A, Prazeres R, Glotin F, Ortega JM, Al-Sawaftah M, de Frutos M (2008). Chemical mapping of the distribution of viruses into infected bacteria with a photothermal method. Ultramicroscopy.

[36] Mayet C, Dazzi A, Prazeres R, Ortega JM, Jaillard D (2010). In situ identification and imaging of bacterial polymer nanogranules by infrared nanospectroscopy. Analyst.

[37] Baldassarre L, Giliberti V, Rosa A, Ortolani M, Bonamore A, Baiocco P (2016). Mapping the amide I absorption in single bacteria and mammalian cells with resonant infrared nanospectroscopy. Nanotechnology.

[38] Deniset-Besseau A, Prater CB, Virolle MJ, Dazzi A (2014). Monitoring TriAcylGlycerols Accumulation by Atomic Force Microscopy Based Infrared Spectroscopy in Streptomyces Species for Biodiesel Applications. J Phys Chem Lett.

[39] Rebois R, Onidas D, Marcott C, Noda I, Dazzi A (2017). Chloroform induces outstanding crystallization of poly(hydroxybutyrate) (PHB) vesicles within bacteria. Anal Bioanal Chem.

[40] Kochan K, Nethercott C, Perez Guaita D, Jiang JH, Peleg AY, Wood BR (2019). Detection of Antimicrobial Resistance-related changes in biochemical composition of Staphylococcus aureus by means of Atomic Force Microscopy-Infrared Spectroscopy. Anal Chem.

[41] Otzen DE, Dueholm MS, Najarzadeh Z, Knowles TPJ, Ruggeri FS (2021). In situ sub-cellular identification of functional amyloids in Bacteria and Archaea by Infrared Nanospectroscopy. Small Methods.

[42] Kochan K, Peleg AY, Heraud P, Wood BR. Atomic Force Microscopy Combined with Infrared Spectroscopy as a Tool to Probe single bacterium Chemistry. J Vis Exp. 2020(163). 10.3791/61728 33016949

[43] Langenberg T, Gallardo R, van der Kant R, Louros N, Michiels E, Duran-Romaña R et al. Thermodynamic and evolutionary coupling between the native and amyloid state of globular proteins. Cell Reports2020. 10.1016/j.celrep.2020.03.076 PMC7175379 32294448

[44] Dos Santos ACVD, Heydenreich R, Derntl C, Mach-Aigner AR, Mach RL, Ramer G (2020). Nanoscale Infrared Spectroscopy and Chemometrics Enable detection of intracellular protein distribution. Anal Chem.

[45] Pachitariu M, Stringer C (2022). Cellpose 2.0: how to train your own model. Nat Methods.

[46] Zack GW, Rogers WE, Latt SA (1977). Automatic measurement of sister chromatid exchange frequency. J Histochem Cytochem.

[47] Raussens V, Waeytens J, Arluison V, Wien F, Marcoleta A (2022). Characterization of bacterial amyloids by Nano-infrared spectroscopy. Bacterial amyloids: methods and protocols.

[48] Kenkel S, Gryka M, Chen L, Confer MP, Rao A, Robinson S (2022). Chemical imaging of cellular ultrastructure by null-deflection infrared spectroscopic measurements. Proc Natl Acad Sci U S A.

[49] Lu F, Jin MZ, Belkin MA (2014). Tip-enhanced infrared nanospectroscopy via molecular expansion force detection. Nat Photonics.

[50] Schwartz JJ, Jakob DS, Centrone A (2022). A guide to nanoscale IR spectroscopy: resonance enhanced transduction in contact and tapping mode AFM-IR. Chem Soc Rev.

[51] Shen Y, Chen A, Wang W, Shen Y, Ruggeri FS, Aime S (2023). The liquid-to-solid transition of FUS is promoted by the condensate surface. Proc Natl Acad Sci U S A.

[52] Ramer G, dos Santos ACV, Zhang Y, Yilmaz U, Lendl B, editors. Image processing as basis for chemometrics in photothermal atomic force microscopy infrared imaging. Advanced Chemical Microscopy for Life Science and Translational Medicine 2023; 2023: SPIE.

[53] McInnes L, Healy J, Saul N, Großberger L. UMAP: Uniform Manifold approximation and projection. J Open Source Softw2018. p. 861.

[54] Simone D, Andrzej S, Angela S, Marco R, Daniele P (2023). Atomic force microscopy as a tool for mechanical characterization at the nanometer scale. Nanomaterials Energy.

[55] Mayet C, Dazzi A, Prazeres R, Allot F, Glotin F, Ortega JM (2008). Sub-100 nm IR spectromicroscopy of living cells. Opt Lett.

[56] Ramer G, Ruggeri FS, Levin A, Knowles TPJ, Centrone A (2018). Determination of Polypeptide Conformation with Nanoscale Resolution in Water. ACS Nano.

[57] Kennedy E, Al-Majmaie R, Al-Rubeai M, Zerulla D, Rice JH (2013). Nanoscale infrared absorption imaging permits non-destructive intracellular photosensitizer localization for subcellular uptake analysis. RSC Adv.

[58] Paluszkiewicz C, Piergies N, Chaniecki P, Rękas M, Miszczyk J, Kwiatek WM (2017). Differentiation of protein secondary structure in clear and opaque human lenses: AFM – IR studies. J Pharm Biomed Anal.

[59] Qamar S, Wang G, Randle SJ, Ruggeri FS, Varela JA, Lin JQ (2018). FUS phase separation is modulated by a molecular chaperone and methylation of Arginine Cation-Pi interactions. Cell.

[60] Zhaliazka K, Kurouski D (2022). Nanoscale characterization of parallel and antiparallel beta-sheet amyloid Beta 1–42 aggregates. ACS Chem Neurosci.

[61] Ruggeri FS, Mannini B, Schmid R, Vendruscolo M, Knowles TPJ (2020). Single molecule secondary structure determination of proteins through infrared absorption nanospectroscopy.

[62] Pancani E, Mathurin J, Bilent S, Bernet-Camard M-F, Dazzi A, Deniset-Besseau A (2018). High-resolution label-free detection of Biocompatible Polymeric nanoparticles in cells. Part Part Syst Charact.

[63] Perez-Guaita D, Kochan K, Batty M, Doerig C, Garcia-Bustos J, Espinoza S (2018). Multispectral Atomic Force Microscopy-Infrared Nano-Imaging of Malaria Infected Red Blood cells. Anal Chem.

[64] Rizevsky S, Kurouski D (2020). Nanoscale Structural Organization of Insulin Fibril Polymorphs revealed by Atomic Force Microscopy-Infrared spectroscopy (AFM-IR). ChemBioChem.

[65] Roman M, Wrobel TP, Paluszkiewicz C, Kwiatek WM (2020). Comparison between high definition FT-IR, Raman and AFM-IR for subcellular chemical imaging of cholesteryl esters in prostate cancer cells. J Biophotonics.

