# Supplementary material for: An end-to-end approach for single-cell infrared absorption spectroscopy of bacterial inclusion bodies: from AFM-IR measurement to data interpretation of large sample sets
Source: J Nanobiotechnology. 2024 Jul 10;22:406. doi: 10.1186/s12951-024-02674-3 (PMC11234752; doi:10.1186/s12951-024-02674-3)
Supplement: Supplementary file 1 — Supplementary Material 1 [file 12951_2024_2674_MOESM1_ESM.docx]

An End-to-End Approach for Single-Cell Infrared Absorption Spectroscopy of Bacterial Inclusion Bodies: From AFM-IR Measurement to Data Interpretation of Large Sample Sets

Supplementary Information

Wouter Duverger^1,2^, Grigoria Tsaka^1,2,3^, Ladan Khodaparast^1,2^, Laleh Khodaparast^1,2^, Nikolaos Louros^1,2^, Frederic Rousseau^1,2,*^ & Joost Schymkowitz^1,2,*^

^1^ Switch Laboratory, VIB-KU Leuven Center for Brain & Disease Research, Herestraat 49, 3000 Leuven, Belgium

^2^ Department of Cellular and Molecular Medicine, KU Leuven, Herestraat 49, box 802, 3000 Leuven, Belgium

^3^ Laboratory for Neuropathology, Department of Imaging and Pathology, KU Leuven, Leuven, Belgium

^*^ Corresponding authors: Frederic Rousseau (frederic.rousseau@kuleuven.be) and Joost Schymkowitz (joost.schymkowitz@kuleuven.be)

Table of Contents

[Methods 2](#_Toc170734407)

[Structured illumination microscopy (SIM) 2](#_Toc170734408)

[Scanning electron microscopy (SEM) 2](#_Toc170734409)

[Transmission electron microscopy (TEM) 3](#_Toc170734410)

[IB purification 3](#_Toc170734411)

[Note S1. Protocol optimisation 4](#_Toc170734412)

[Note S2. AFM-IR quality control 5](#_Toc170734413)

[Note S3. List of measurements and cantilevers used 5](#_Toc170734414)

[Note S4. Alternative analysis of PLL Frequency 9](#_Toc170734415)

[Note S5. Second derivative spectra of heat shock IBs 10](#_Toc170734416)

[Note S6. Validations of AFM-IR results 10](#_Toc170734417)

[Note S7. Description of IB properties 15](#_Toc170734418)

[References 16](#_Toc170734419)

# Methods

## Structured illumination microscopy (SIM)

An overnight culture was diluted in saline to 2 MacFarland units. 500 µL of culture was washed twice by centrifuging at 4300 g for 2 min and replacing the supernatant with 500 µL saline and once with 100 µL saline. pFTAA was added to a final concentration of 5 µM and the sample was incubated at 49°C for 1 hour, after the sample was spun down again and 5 µL was spotted on a slide and imaged in a Nikon Ti2 N-SIM S microscope at 488 nm excitation.

## Scanning electron microscopy (SEM)

From sample 4 of **Figure 2** (see **Table S1**), we made another section and deposited it on a silicon wafer, which was mounted on an SEM stub with silver paint. The sample was imaged on a Zeiss VP Sigma SEM first by recording the backscattered electrons with a Gatan Onpoint BSE-detector. After adding a layer of 8 nm of chrome with a Leica ACE600 coating unit, the sample was imaged again with secondary electron (SE)-detector for topographical information.

## Transmission electron microscopy (TEM)

From one of the heat shock treated samples of **Figure 4**, we produced two consecutive sections and deposited one on a silicon wafer for AFM-IR and one on a TEM support grid (Agar Scientific, AGS162-4). The AFM-IR sample was imaged according to the protocol detailed in this paper and the other sample was imaged in a JEOL JEM 1400 microscope equipped with an Olympus Quemesa detector, operated at 80 kV.

## IB purification

On three different days, we prepared samples from overnight cultures exposed to 1 hour at 49°C then 1 hour at 37°C (recovery), 1 hour at 37°C and 1 hour at 49°C (heat shock), or 2 hours at 37°C. These samples were spun down (12000 g, 4°C, 15 min), resuspended in 200 µL lysis buffer (50 mM Tris-Cl, pH 8, 1 mM EDTA, 100 mM NaCl), supplemented with lysozyme (to 10 mg/mL) and PMSF (150 µL added per mL of buffer, from 100 mM stock in isopropanol) and incubated for 30 min at 37°C while shaking. NP-40 was supplemented to 1 vol% and incubated at 4°C for 30 min. 3 µL of DNase I (1 mg/mL) and 3 µL of MgSO_4_ (1 M) were added and incubated at 37°C for 30 min. The sample was spun down and resuspended in 200 µL lysis buffer supplemented with Triton X-100 to 0.5 vol%. Samples were washed with 200 µL sterile PBS. Then they were spun down, the supernatant was discarded, and the pellets were stored at -80°C.

The pellets were later resuspended in 100 µL ddH_2_O, of which 10 µL was deposited on a gold substrate (Platypus Technologies, AU.1000.SWTSG). After 10 min, the substrate was rinsed three times, excess liquid was blotted away, and the sample was left to air-dry before gluing onto a sample disc and imaging with AFM-IR.

# Note S1. Protocol optimisation

Raw height maps underlying **Figure 1E-F** are shown in **Figure S1**.

**Figure S1.** Consecutive AFM heightmap images of amyloid fibrils on atomically flat gold substrate. Time passed between acquisition of each image and the first image is noted on the top left of each image.

To measure to what extent IR amplitude signals may be caused by differences in mechanical contact, we collected AFM-IR datasets at 1625 and 1650 cm⁻¹, wavenumbers used throughout the experiments, and compared them to an AFM-IR dataset at 1450 cm⁻¹ **(Figure S2)**. Due to the low IR Amplitude measured, the PLL was rather unstable, but no significant contrast was observed, except at membrane locations and a dust particle at the bottom of the image.


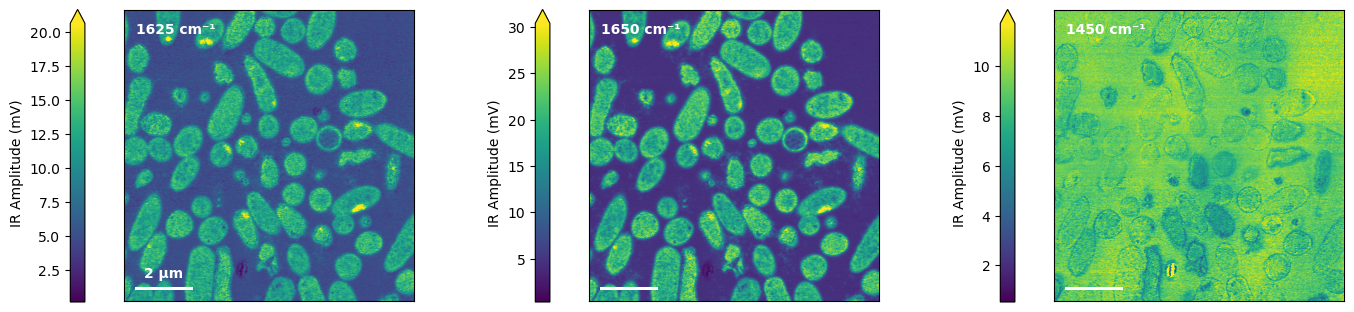


**Figure S2.** IR Amplitude images of ultrathin section of bacteria embedded in epoxy resin at 1625, 1650 and 1450 cm⁻¹.

# Note S2. AFM-IR quality control

To assess data quality, the raw data from all AFM-IR datasets and spectra analysed in this paper are presented in the following documents on figshare:

- **Quality control of AFM-IR datasets.** Raw data for all AFM datasets in this paper, including height (flattened), deflection (with respect to setpoint), IR Amplitude, PLL Frequency, and IR Phase. Displays vertical and horizontal profiles in all available scanning directions (trace: blue, retrace: orange). <https://doi.org/10.6084/m9.figshare.25398682.v2>
- **Additional File 2 (PDF) Quality control of AFM-IR spectra.** Shows the localization, raw IR Amplitude data, PLL Frequency and IR Phase (if available) for all spectra of epoxy-embedded samples in this paper. <https://doi.org/10.6084/m9.figshare.25398583.v2>

# Note S3. List of measurements and cantilevers used

**Table S1.** Sample details in **Figure 2**. All samples were prepared from different cultures.

| **Sample number** | **Sample id** | **Preparation date** |
| --- | --- | --- |
| 1 | M1A | 2023/03/01 |
| 2 | M1B | 2023/03/01 |
| 3 | M1C | 2023/03/01 |
| 4 | J10 | 2022/01/10 |
| 5 | O21 | 2021/10/21 |

**Table S2.** Cantilevers used in **Figure 2**.

| **Measurement date** | **Sample** | **Technical repeat** | **Cantilever** |
| --- | --- | --- | --- |
| 2023/03/07 | M1A | 1 | TB007I |
| 2023/03/07 | M1B | 1 | TB007I |
| 2023/03/08 | M1C | 1 | TB007I |
| 2023/03/08 | M1A | 2 | TB007J |
| 2023/03/09 | M1B | 2 | TB007J |
| 2023/03/09 | M1C | 2 | TB007J |
| 2023/03/10 | O21 | 2 | TB007J |
| 2023/03/10 | J10 | 2 | TB007J |
| 2023/03/13 | M1A | 3 | TB008A |
| 2023/03/14 | M1B | 3 | TB008A |
| 2023/03/14 | M1C | 3 | TB008A |
| 2023/03/14 | O21 | 3 | TB008A |
| 2023/03/15 | J10 | 3 | TB008A |
| 2023/03/19 | M1A | 4 | TB008B |
| 2023/03/20 | M1B | 4 | TB008B |
| 2023/03/20 | M1C | 4 | TB008B |
| 2023/03/20 | O21 | 4 | TB008B |
| 2023/03/21 | J10 | 4 | TB008B |
| 2024/06/03 | M1A | 5 | TB012D |
| 2024/06/03 | M1B | 5 | TB012D |
| 2024/06/04 | M1C | 5 | TB012D |
| 2024/06/04 | O21 | 5 | TB012D |
| 2024/06/05 | J10 | 5 | TB012D |

**Table S3.** Cantilevers used in **Figure 4.**

| **Measurement date** | **Biological replicate** | **Condition** | **Cantilever** |
| --- | --- | --- | --- |
| 2024/01/03 | A | B | TB010H |
| 2024/01/04 | A | H | TB010H |
| 2024/01/05 | A | P | TB010H |
| 2024/01/08 | B | P | TB010I |
| 2024/01/09 | B | B | TB010I |
| 2024/01/10 | B | H | TB010I |
| 2024/01/10 | C | B | TB010J |
| 2024/01/15 | C | P | TB010J |
| 2024/01/15 | C | H | TB010J |

**Table S4.** Cantilevers used in **Figure 5**. Same-series samples were prepared at the same time.

| **Measurement** | **Series** | **Biological replicate** | **Time post shock** | **Cantilever** |
| --- | --- | --- | --- | --- |
| 2023/04/04 | Apr | A | -1 | TB008D |
| 2023/04/05 | Apr | A | 0 | TB008D |
| 2023/04/05 | Apr | A | 1 | TB008D |
| 2023/04/06 | Apr | B | -1 | TB008D |
| 2023/04/06 | Apr | B | 0 | TB008D |
| 2023/04/07 | Apr | B | 1 | TB008D |
| 2023/04/07 | Apr | C | -1 | TB008D |
| 2023/04/08 | Apr | C | 0 | TB008D |
| 2023/04/09 | Apr | C | 1 | TB008D |
| 2023/07/19 | Jul | A | -1 | TB008J |
| 2023/07/20 | Jul | A | 0 | TB008J |
| 2023/07/20 | Jul | A | 0.5 | TB008J |
| 2023/07/21 | Jul | A | 1 | TB008J |
| 2023/07/23 | Jul | A | 2 | TB008J |
| 2023/07/12 | Jul | B | -1 | TB008J |
| 2023/07/13 | Jul | B | 0 | TB008J |
| 2023/07/13 | Jul | B | 0.5 | TB008J |
| 2023/07/13 | Jul | B | 1 | TB008J |
| 2023/07/14 | Jul | B | 2 | TB008J |
| 2023/07/14 | Jul | C | -1 | TB008J |
| 2023/07/17 | Jul | C | 0 | TB008J |
| 2023/07/18 | Jul | C | 0.5 | TB008J |
| 2023/07/18 | Jul | C | 1 | TB008J |
| 2023/07/18 | Jul | C | 2 | TB008J |
| 2023/11/14 | Nov | A | -1 | TB009F |
| 2023/11/15 | Nov | A | 0 | TB009F |
| 2023/11/15 | Nov | A | 0.5 | TB009F |
| 2023/11/16 | Nov | A | 1 | TB009H |
| 2023/11/16 | Nov | A | 2 | TB009H |
| 2023/11/19 | Nov | B | -1 | TB009J |
| 2023/11/19 | Nov | B | 0 | TB009J |
| 2023/11/21 | Nov | B | 0.5 | TB010B |
| 2023/11/22 | Nov | B | 1 | TB010B |
| 2023/11/26 | Nov | B | 2 | TB010C |
| 2023/11/26 | Nov | C | -1 | TB010C |
| 2023/11/27 | Nov | C | 0 | TB010D |
| 2023/11/28 | Nov | C | 0.5 | TB010D |
| 2023/11/29 | Nov | C | 1 | TB010D |
| 2023/11/30 | Nov | C | 2 | TB010D |

# Note S4. Alternative analysis of PLL Frequency

For the image-based analysis of IB stiffness, this paper focuses uniquely on the difference between the average PLL Frequency of the IB and the cytoplasm. This approach is less sensitive to technical artifacts such as PLL Frequency drift and instability. **Figure S3 (Left)** shows that considering only the mean IB PLL Frequency introduces significant differences between repeat measurements (the figure reports for each sample the p-value of an ANOVA on all data points, excluding repeat 2).

This problem is partially addressed by Chen et al. (1). Essentially, they perform a fit of seven Gaussians to the PLL Frequency distribution of an image and classify each pixel as belonging to one of the seven populations. This is a way of standardizing PLL data, allowing for changes in the location and spread of these distributions. Because the epoxy pixels provide a clear reference in each sample, we performed a similar analysis by calculating the mean and standard deviation of the PLL Frequency of all epoxy pixels on each row of the image. We then applied a rolling mean on those statistics and used the results to calculate a PLL z-score for all pixels in the image see **Figure S3 (Right)**. This measure is less sensitive to technical variability in terms of the IB PLL z-score, but most samples still exhibit statistically significant variations in the cytoplasm PLL z-score.


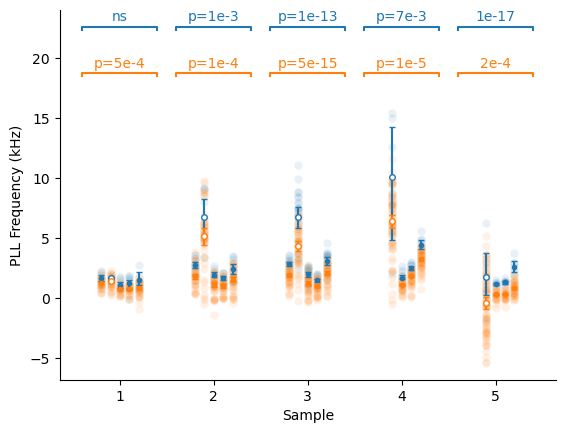

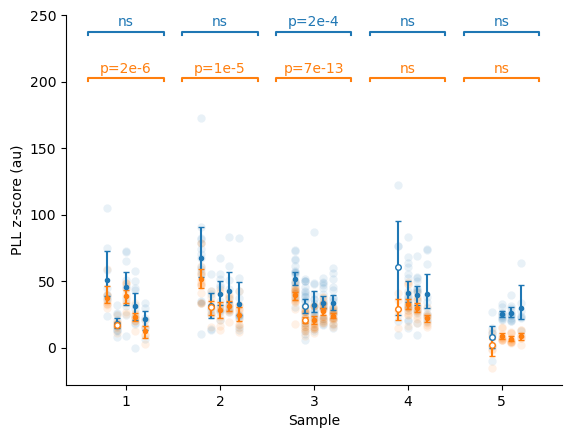


**Figure S3.** Other means of analysing the PLL Frequency. **(Left)** The mean PLL Frequency and **(Right)** the mean PLL z-score of each IB (blue) and cytoplasm (orange). Statistical annotations report the adjusted p-value of an ANOVA on all data points for that sample, except repeat 2, to assess the technical variability. Repeat 2, an outlier, is indicated by hollow markers. Error bars represent a 95% confidence interval, by bootstrap.

# Note S5. Second derivative spectra of heat shock IBs

Second derivative analysis (**Figure S4**) reveals three β-sheet-related peaks that differentiate between the two kinds of spectra. The first, at 1641 cm⁻¹ is only present in the cytoplasm. The peaks in IBs are found at 1636 cm⁻¹ (intramolecular) and 1622 cm⁻¹ (intermolecular) (2).


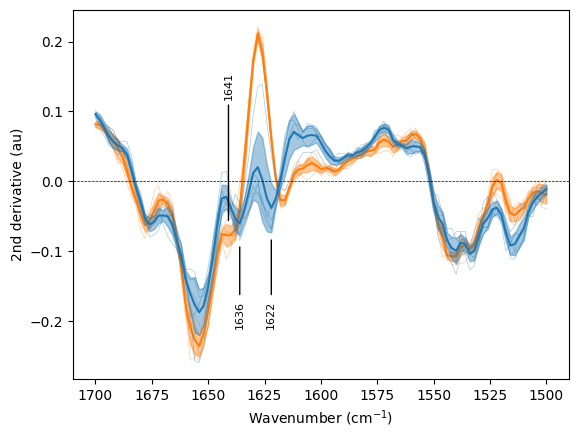


**Figure S4.** Second derivative spectra of IBs (blue) and cytoplasma (orange) in the heat shock recovery dataset, with annotated relevant peaks.

# Note S6. Validations of AFM-IR results

To confirm the results in **Figure 5**, we purified IBs from overnight cultures that spent 2h at 37°C (baseline, B), one that spent 1h at 37°C, then 1h at 49° (heat shock, H), and one that spent 1h at 49°C, then 1h at 37° (recovery, R). These were deposited on a gold substrate and imaged them with AFM-IR. The data is shown in **Figure S5**. Spectra from gold substrate are not shown. To process IB spectra, first the average gold spectrum per sample was calculated and the peaks due to water absorption were estimated. Then, the IB spectra were normalized to the amide 1 peak, divided by the water peak spectrum, and finally the gold spectrum was subtracted.


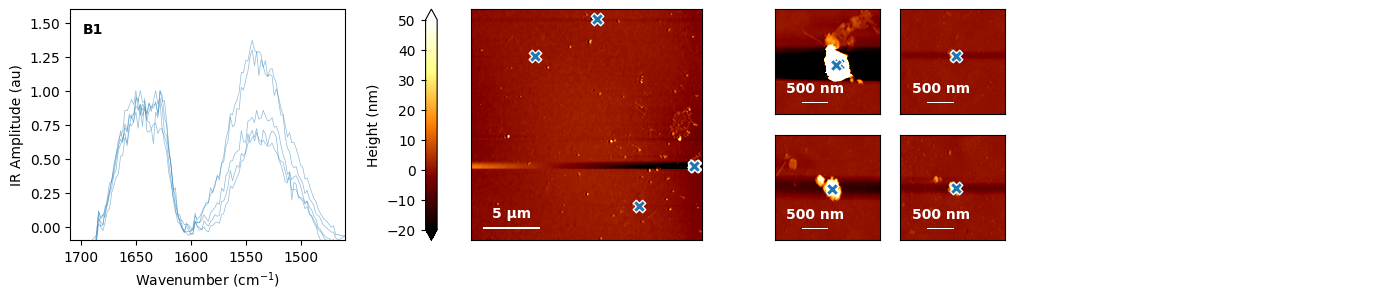


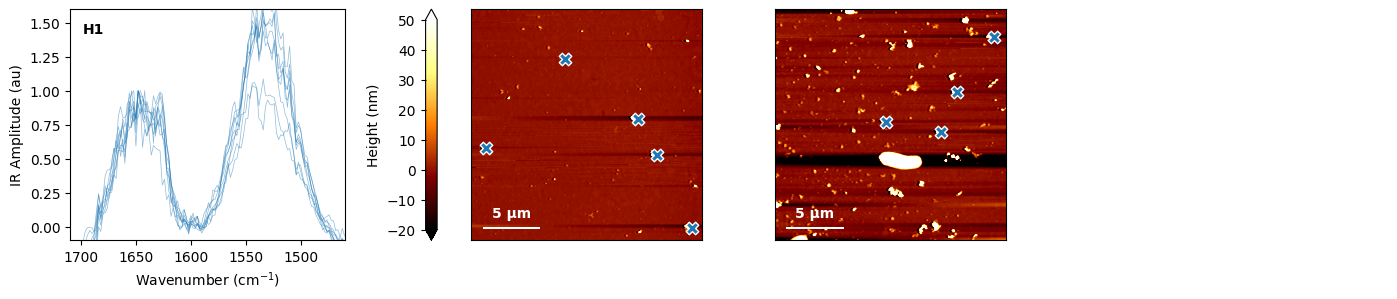

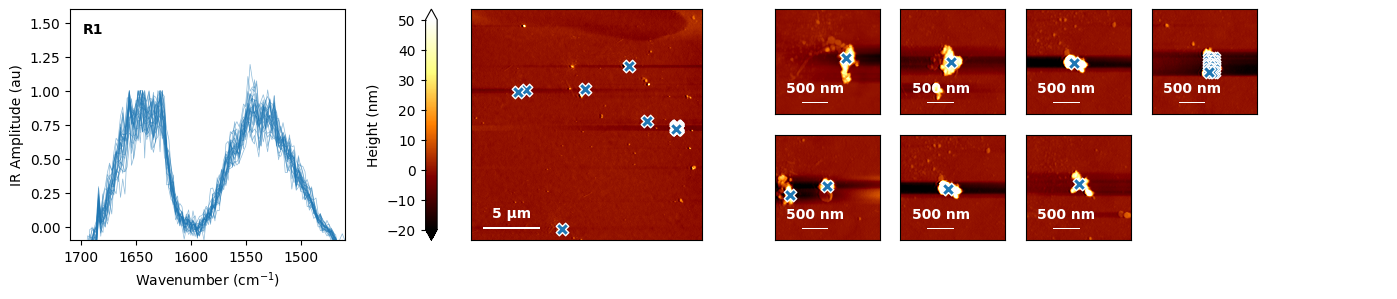


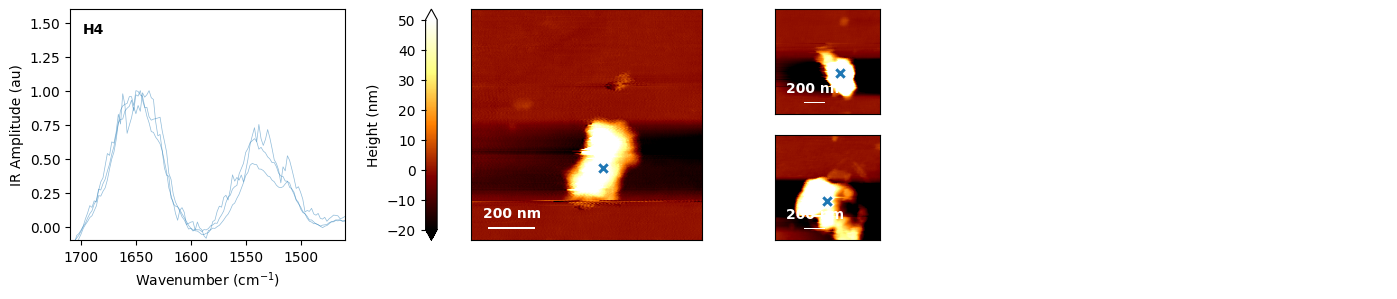

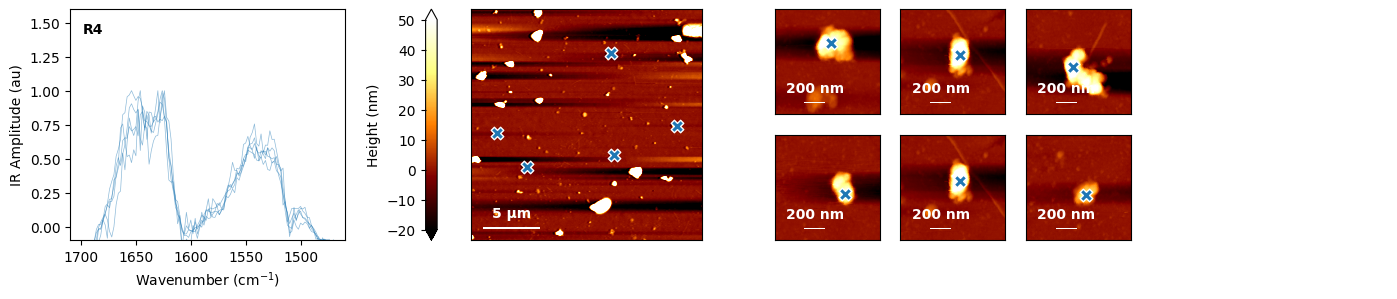

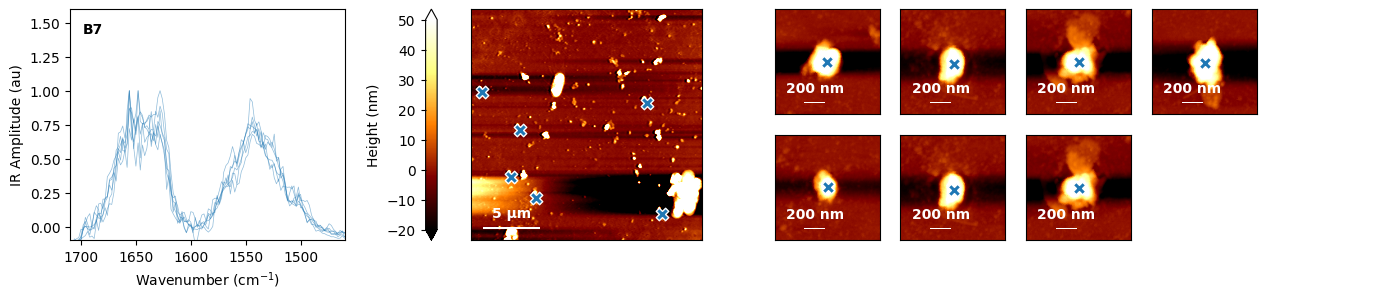

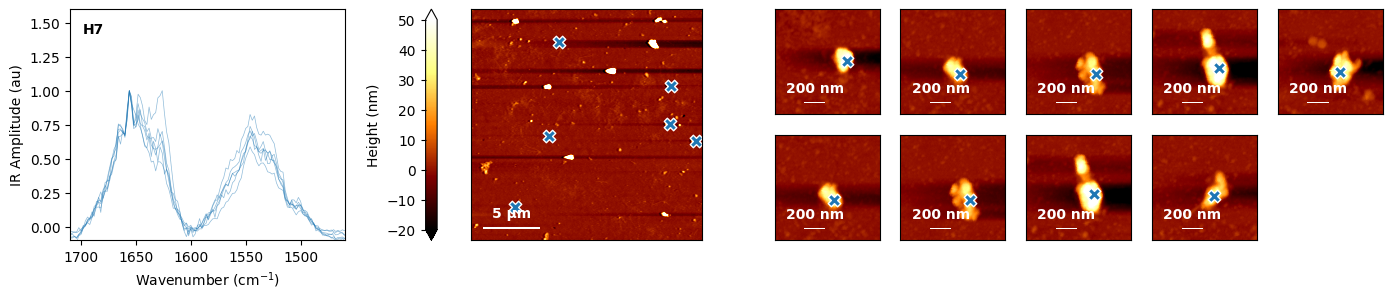

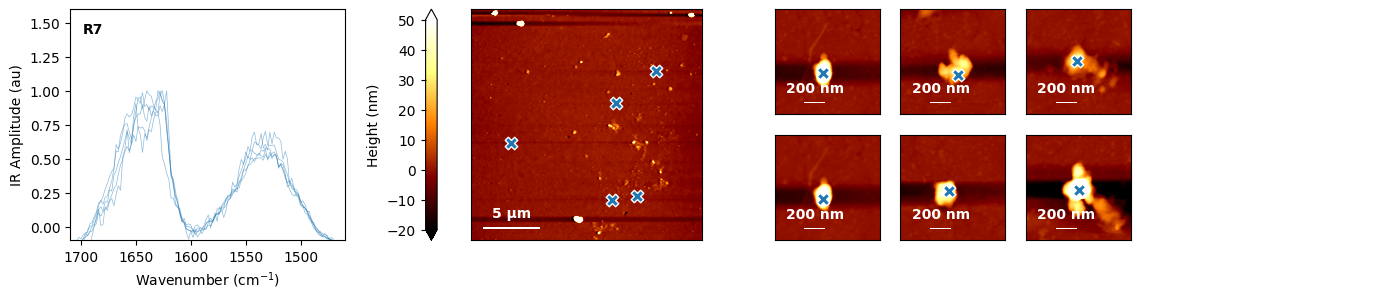


**Figure S5.** AFM-IR data of purified inclusion bodies. For each sample of IBs, normalized spectra are plotted, along with their localization.

A quantification of the beta/alpha ratio (integral from 1615 to 1635 cm⁻¹ divided by one from 1650 to 1670 cm⁻¹) is plotted in **Figure S6**. Because of the low sample size after averaging to one spectrum per sample, we performed a Mann-Whitney U-test to compare the means between the different conditions. The beta-to-alpha ratio of baseline IBs was slightly different from heat shock (p_adj=.049) and not distinguishable from recovery (p_adj=.4), but heat shock IBs had a significantly lower ratio than recovery IBs (p=.0001).


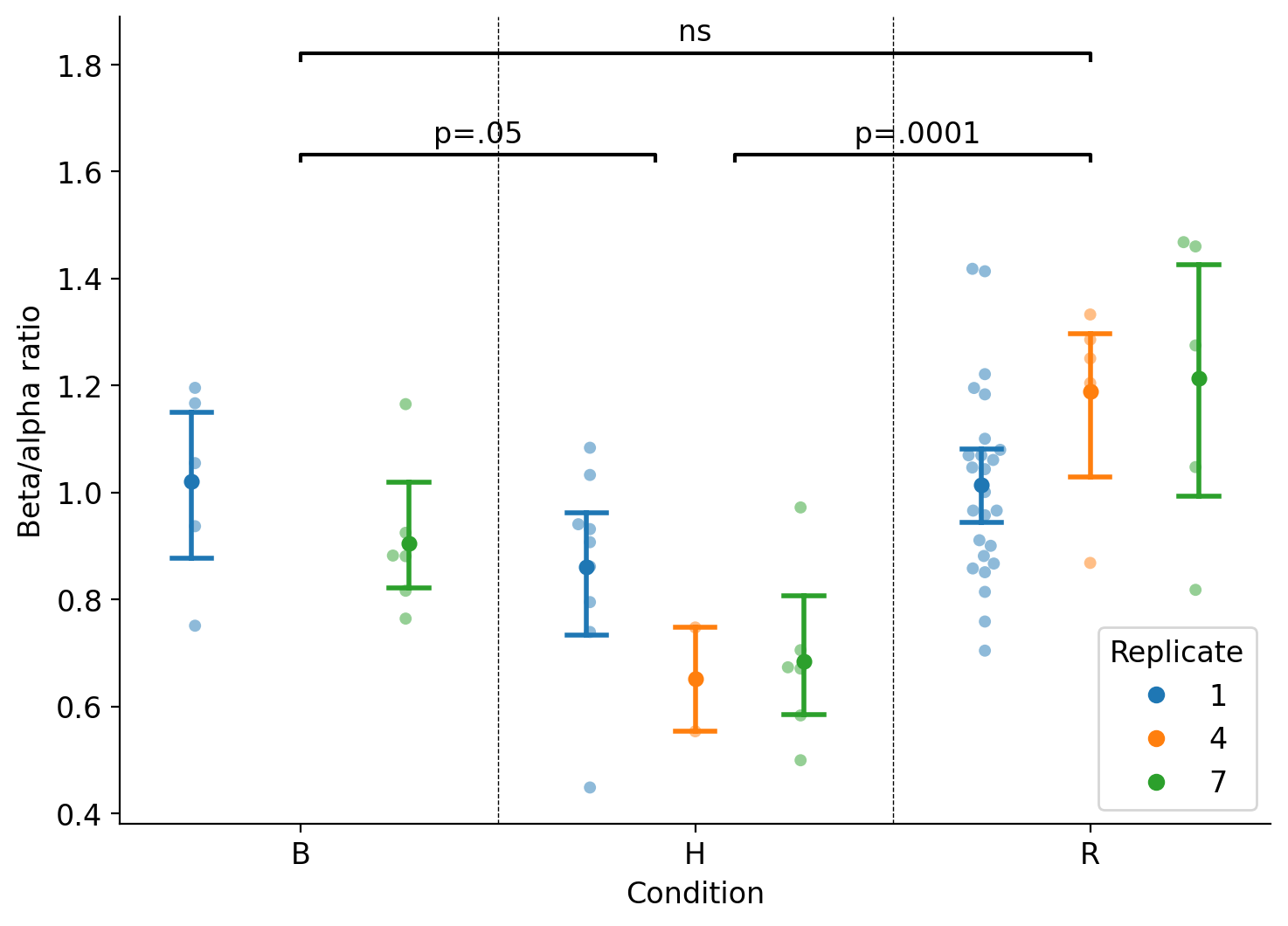


**Figure S6.** Quantification of beta-to-alpha ratio in IB spectra.

Additionally, we performed SEM on a section of non-stressed resin-embedded bacteria after AFM-IR imaging, shown in **Figure S7**. There are some small spontaneous inclusions in the sample, but they are invisible in the electron density map (compared to our previous work (3)). The surface topology in AFM matches SEM. There is a small amount of surface wear visible at the borders of where the AFM-IR image was taken. AFM-IR data and TEM microscopy on consecutive sections reveals a striking correlation between those regions with high beta sheet content and high electron density, characteristic of IBs, see **Figure S8**. Finally, we confirmed the amyloid-like nature of IBs by staining with pFTAA, see **Figure S9.**


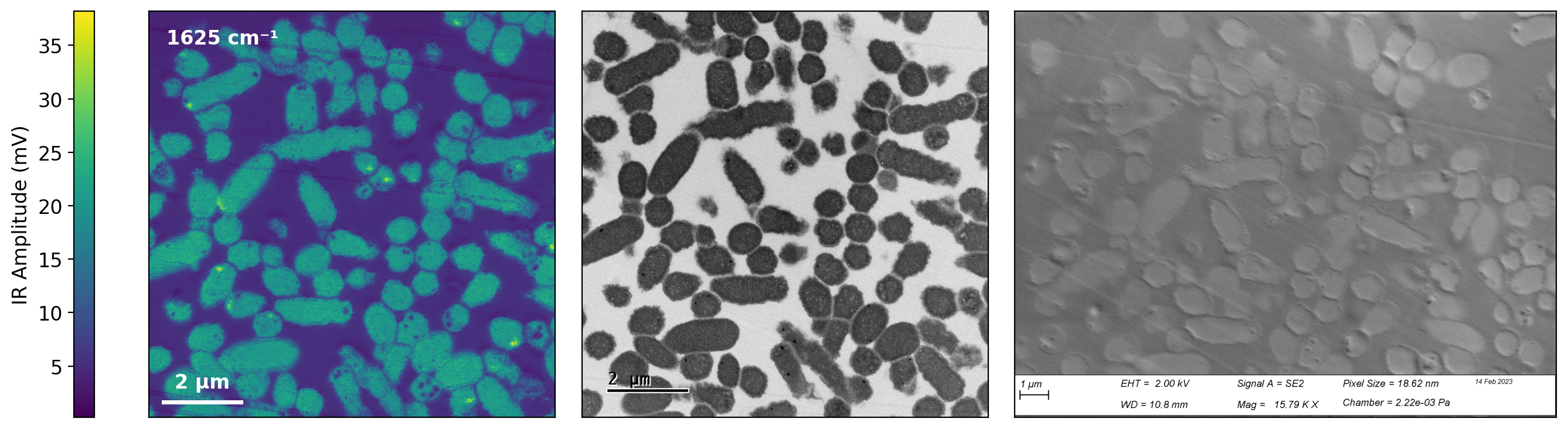


**Figure S7.** Correlative AFM-IR and SEM. **(Left)** AFM-IR image of non-stressed cells at 1625 cm⁻¹ (beta sheets). **(Middle)** SEM backscattering electron image showing electron density. **(Right)** SEM secondary electron image showing surface topology of the sample.


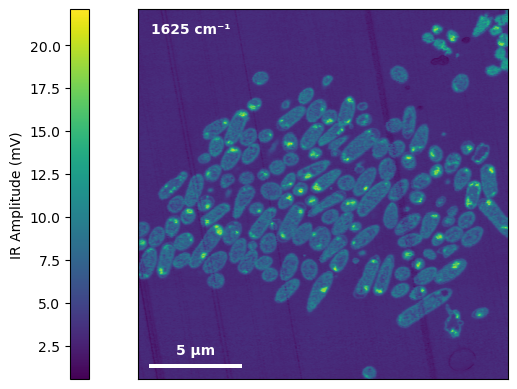

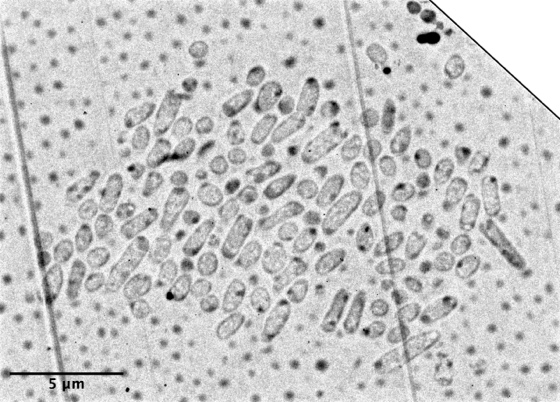


**Figure S8. (Left)** IR Amplitude image of thin section of bacteria embedded in epoxy post heat shock treatment and **(Right)** TEM image of consecutive thin section, reflected and rotated to match the AFM-IR image.


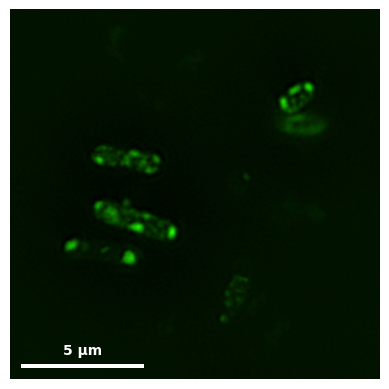


**Figure S9.** SIM micrograph of pFTAA-stained bacteria after heat shock.

# Note S7. Description of IB properties

**Table S5.** The following properties are recorded for every IB, in order of **Figure 6C**:

| **Parameter name** | **Unit** | **Description** |
| --- | --- | --- |
| beta_ratio_cytoplasm | - | Ratio of IR Amplitude at 1625 to 1650 cm⁻¹, averaged over the cytoplasm¹ |
| beta_ratio_ib | - | Ratio of IR Amplitude at 1625 to 1650 cm⁻¹, averaged over the IB |
| roughness_cytoplasm | nm | Standard deviation of measured height values over cytoplasm¹ |
| height_cytoplasm | nm | Measured height values averaged over cytoplasm¹ |
| height_ib | nm | Measured height values averaged over the IB |
| roughness_ib | nm | Standard deviation of measured height values within the IB |
| cell_fraction_ib_area | - | Proportion of the cell² area taken up by IBs |
| ib_area | µm² | Size of the IB |
| ib_eccentricity | - | Mathematical eccentricity of the IB (0 is perfectly circular, and higher is more elliptical) |
| beta_enrichment | - | Ratio of beta_ratio_ib over beta_ratio_cytoplasm |
| iramp_1625_ib | mV | Average IR Amplitude at 1625 cm⁻¹ within the IB |
| iramp_1650_ib | mV | Average IR Amplitude at 1650 cm⁻¹ within the IB |
| iramp_1625_cytoplasm | mV | Average IR Amplitude at 1625 cm⁻¹ within the cytoplasm¹ |
| iramp_1650_cytoplasm | mV | Average IR Amplitude at 1650 cm⁻¹ within the cytoplasm¹ |
| pll_difference | kHz | Difference in average PLL Frequency between the IB and the cytoplasm¹ |
| pll_ib | kHz | Average PLL Frequency within the IB |
| cell_orientation | deg | Angle between polar axis of the cell² and a horizontal line |
| polar_projection_ib | - | Location of the IB with respect to the polar axis (-1 is left-hand pole, +1 is right-hand pole) |
| pll_cytoplasm | kHz | Average PLL Frequency within the cytoplasm¹ |
| polar_proximity_ib | - | Absolute value of polar_projection_ib (0 is central, 1 is polar) |
| cell_num_ibs | - | The number of IBs in the cell² |
| cell_area | µm² | Size of the cell² |
| cell_eccentricity | - | Eccentricity of the cell² |

¹ the area of the cell containing the IB, excluding it and other IBs
² the area of the cell containing the IB, including it and other IBs

# References

1. Chen J, Kim J, Shao W, Schlecht SH, Baek SY, Jones AK, et al. An Anterior Cruciate Ligament Failure Mechanism. The American Journal of Sports Medicine. 2019;47(9):2067-76.

2. Zandomeneghi G, Krebs MR, McCammon MG, Fandrich M. FTIR reveals structural differences between native beta-sheet proteins and amyloid fibrils. Protein Sci. 2004;13(12):3314-21.

3. Khodaparast L, Khodaparast L, Gallardo R, Louros NN, Michiels E, Ramakrishnan R, et al. Aggregating sequences that occur in many proteins constitute weak spots of bacterial proteostasis. Nat Commun. 2018;9(1):866.
